# Supplementary material for: Expression of the Z Variant of α1-Antitrypsin Suppresses Hepatic Cholesterol Biosynthesis in Transgenic Zebrafish
Source: Int J Mol Sci. 2023 Jan 27;24(3):2475. doi: 10.3390/ijms24032475 (PMC9917206; doi:10.3390/ijms24032475)
Supplement: Supplementary file 1 [file ijms-24-02475-s001.zip › ijms-2172606-supplementary.pdf]

Supplementary Figures

Unprocessed gel images/data files are available at https://figshare.com/s/a7fe6149482d21aabf3a

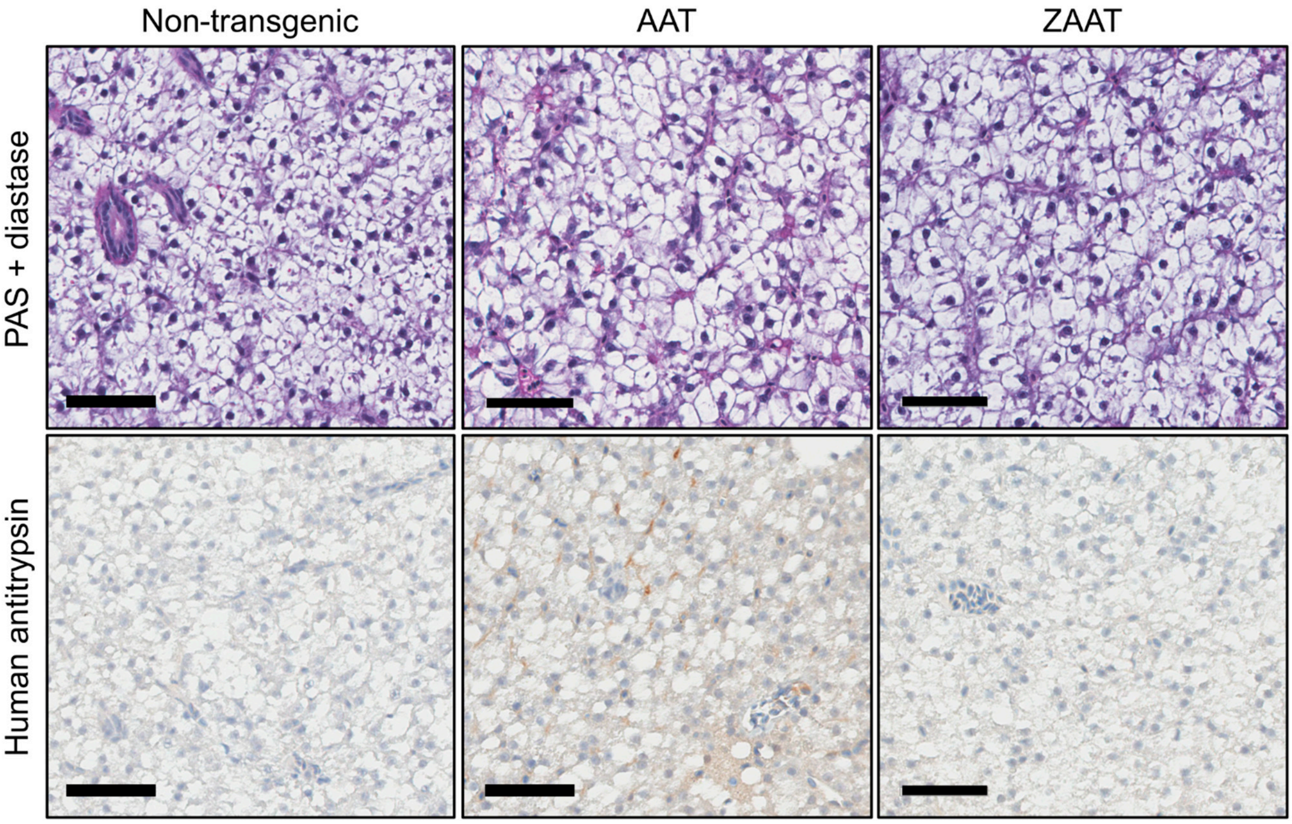

Figure S1. Absence of ZAAT-containing inclusions in transgenic zebrafish liver.
Periodic acid-Schiff (PAS) staining with diastase (top panels) and immunohistochemical staining using a polyclonal guinea pig antibody against human α1-antitrypsin (bottom panel) was performed on 3 μm thick paraffin liver sections from non-transgenic (left panels), AAT-transgenic (middle panels) and ZAAT-transgenic (right panels) adult zebrafish. Scale bar represents 50 μm.

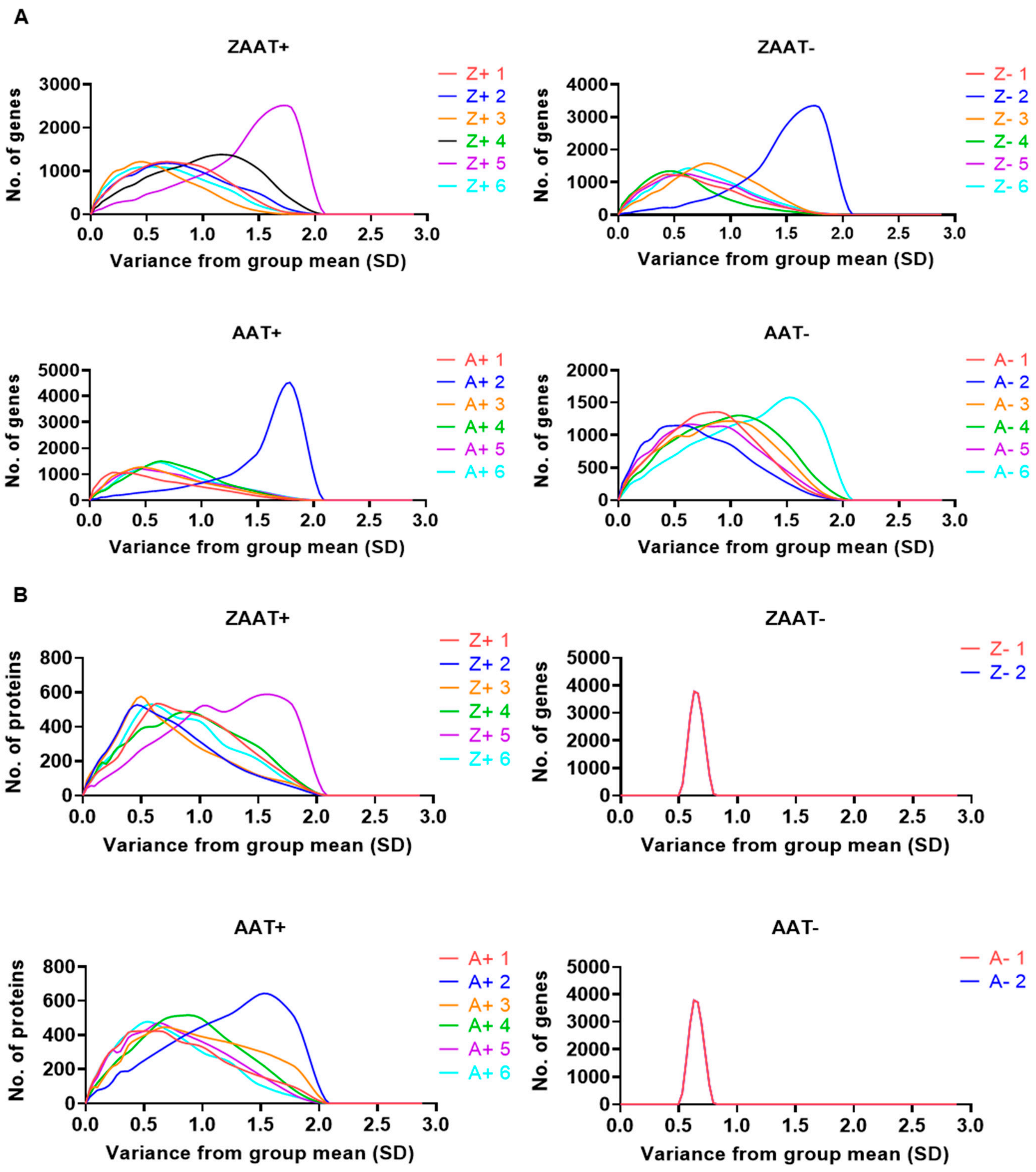

Figure S2. Variation analysis identifies outlier in bioinformatic experiments.

RPKM from RNA-seq and protein counts from LCMS/MS were converted into Z-scores. The number of genes/proteins is plotted against the absolute values of Z-score. Variation analysis of **a)** RNA-seq data identified 1 out of 6 fish in each group (Z+ 5, Z- 2, A+ 2 and A- 6) as outlier, and in **b)** LCMS data, one transgenic fish was identified as outlier in each transgenic group (A+ 2, Z+ 5), consistent RNA-seq. No outlier was identified from non-transgenic sibling groups due to small sample size. These samples were excluded in the downstream differential expression analysis.

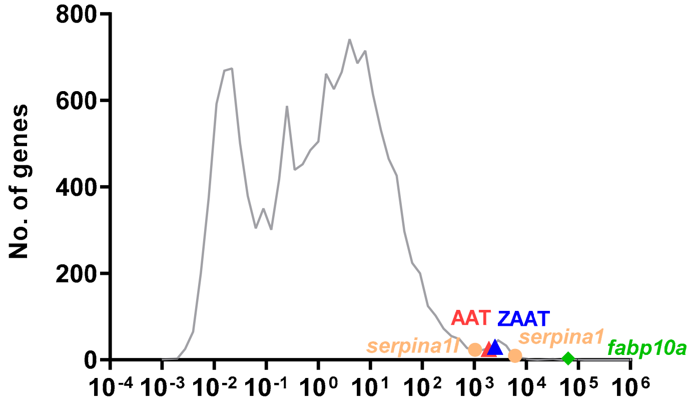

Figure S3. Comparison of transgene expression to endogenous antitrypsin.
Frequency distribution of protein-coding transcripts in zebrafish livers from RNA-seq data, averaging RPKM for each gene from all fish across the four genotype groups. The red triangle indicates the expression of AAT and blue triangle indicates the expression of the ZAAT transgene.

	Proteomics		Transcriptomics	
Student's t-test	Min	Max	Min	Max
Z+ vs. A+	0.000	0.050	0.000	0.010
Z+ vs. [A+Z+]	0.000	0.250	0.000	0.010
Z+ vs. Z-	0.000	0.250	0.000	0.010
Z- vs. A-	0.050	1.000	0.050	1.000
[FDR] threshold	0.250		0.010	
Differential exp genes	77		435	
% of total peptide/genes	1.74%		1.54%	

Figure S4. Statistical parameters used dysregulation expression analysis

Summary of Analysis - Analysis - Data - Human gene filtered 2(1)

Top Canonical Pathways

Name	p-value	Overlap
Superpathway of Cholesterol Biosynthesis	2.06E-14	44.4 % 12/27
Cholesterol Biosynthesis I	1.18E-09	53.8 % 7/13
Cholesterol Biosynthesis II (via 24,25-dihydrolanosterol)	1.18E-09	53.8 % 7/13
Cholesterol Biosynthesis III (via Desmosterol)	1.18E-09	53.8 % 7/13
Superpathway of Geranylgeranyldiphosphate Biosynthesis I (via Mevalonate)	8.19E-06	31.2 % 5/16

Top Upstream Regulators

Upstream Regulator	p-value of overlap	Predicted Activation
SH3TC2	1.16E-12	
SREBF2	7.25E-10	Inhibited
SCAP	1.45E-09	Inhibited
SIRT2	2.83E-08	Inhibited
pitavastatin	6.33E-08	Inhibited

Figure S5. Ingenuity pathway analysis of RNA-seq data.

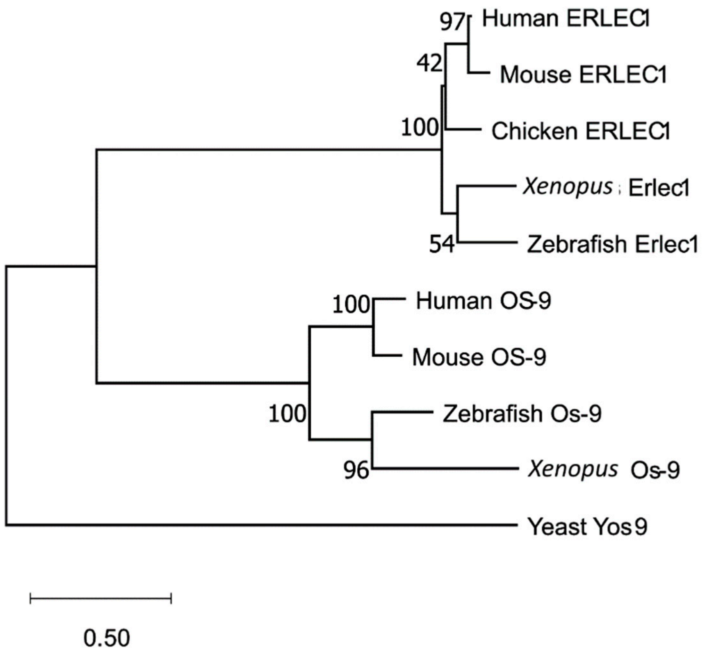

Figure S6. Phylogenetic analysis of ER lectins.

ERLEC1 and OS-9 protein sequences from human, mouse, chicken, *Xenopus*, zebrafish, and yeast were aligned using Clustal-W. Evolutionary history was inferred by using the Maximum Likelihood method and Jones et al. w/freq. model. The tree was calculated from the alignment using Molecular Evolutionary Genetics Analysis software, version 10 (MEGA X). The percentage of trees in which the associated taxa clustered together in the bootstrap test (1000 replicates) are shown next to the branches. Initial tree(s) for the heuristic search were obtained automatically by applying Neighbor-Join and BioNJ algorithms to a matrix of pairwise distances estimated using the Jones-Taylor-Thornton (JTT) model. Scale bar refers to an evolutionary distance (number of substitutions per site) of 0.5.

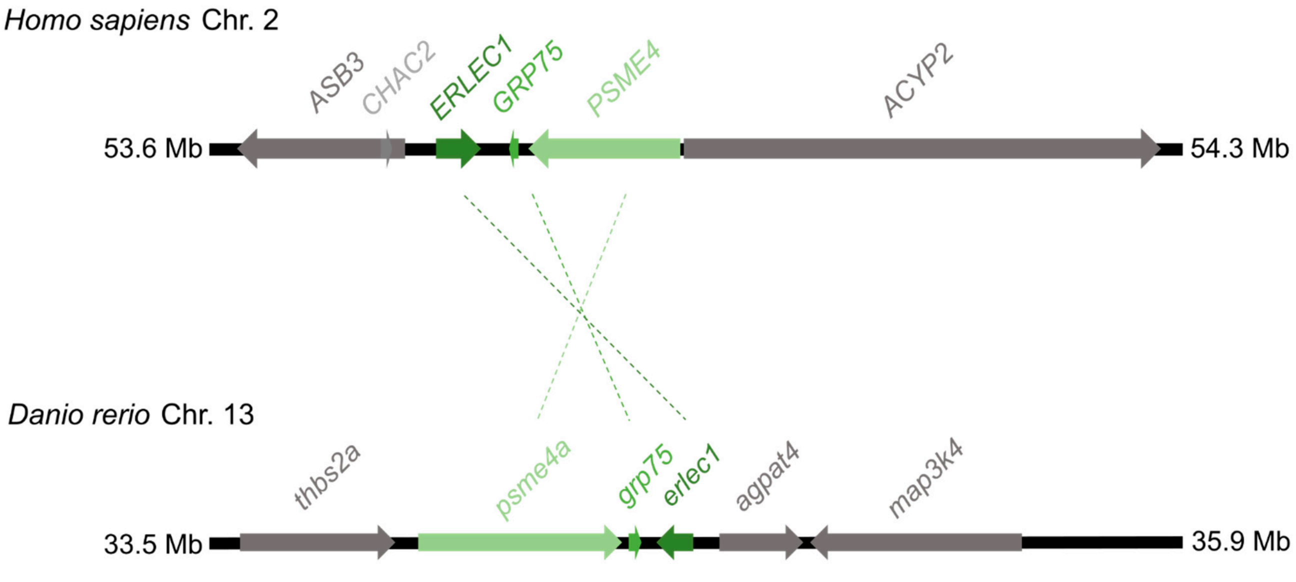

Figure S7. Synteny relationships of *erlec1* gene loci in human and zebrafish.
Mapped genomes of *Homo sapiens* and *Danio rerio* were used to identify conserved gene neighbours of *erlec1*.

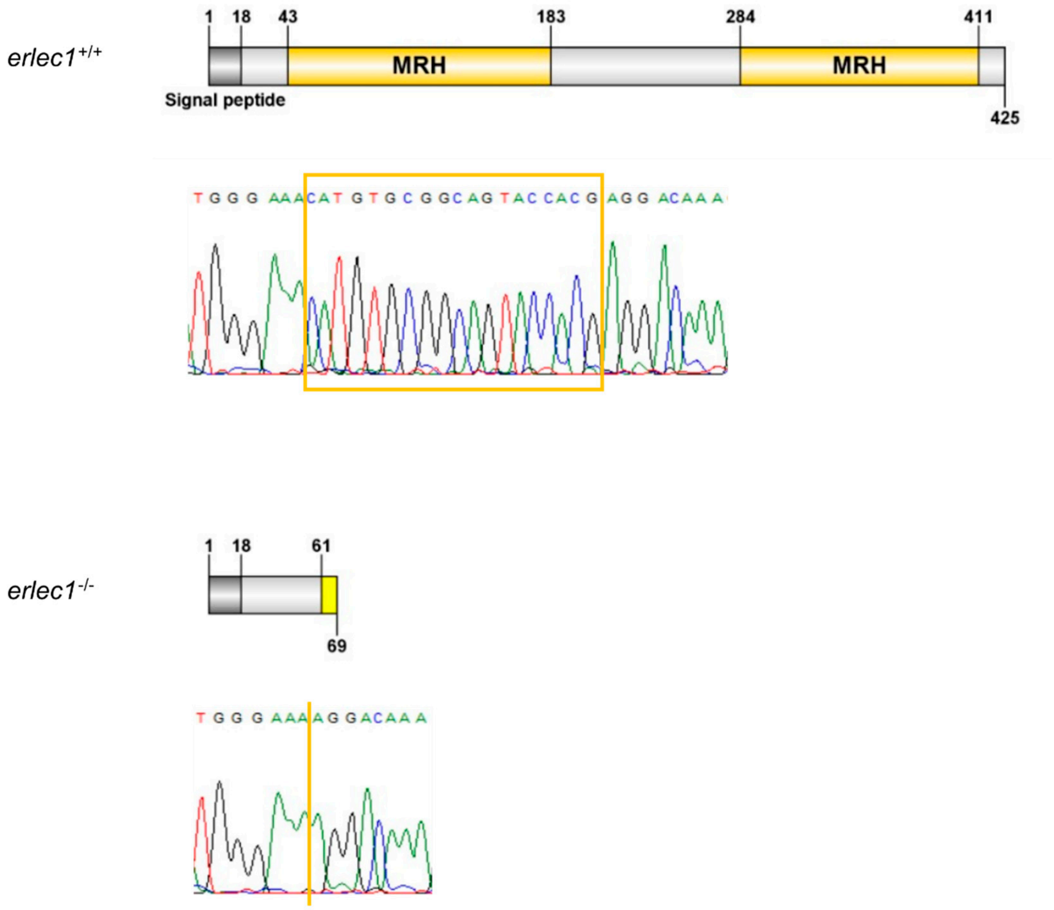

Figure S8. Sequence validation of *erlec1* mutant.
Sequencing of generation F2 homozygotes confirmed a 19 bp deletion at nucleotide positions 184-202 (outlined in yellow), resulting in a frameshift that added 8 amino acids (light yellow) from position 61 before a stop codon. MRH = mannose-6-phosphate receptor homology domain.

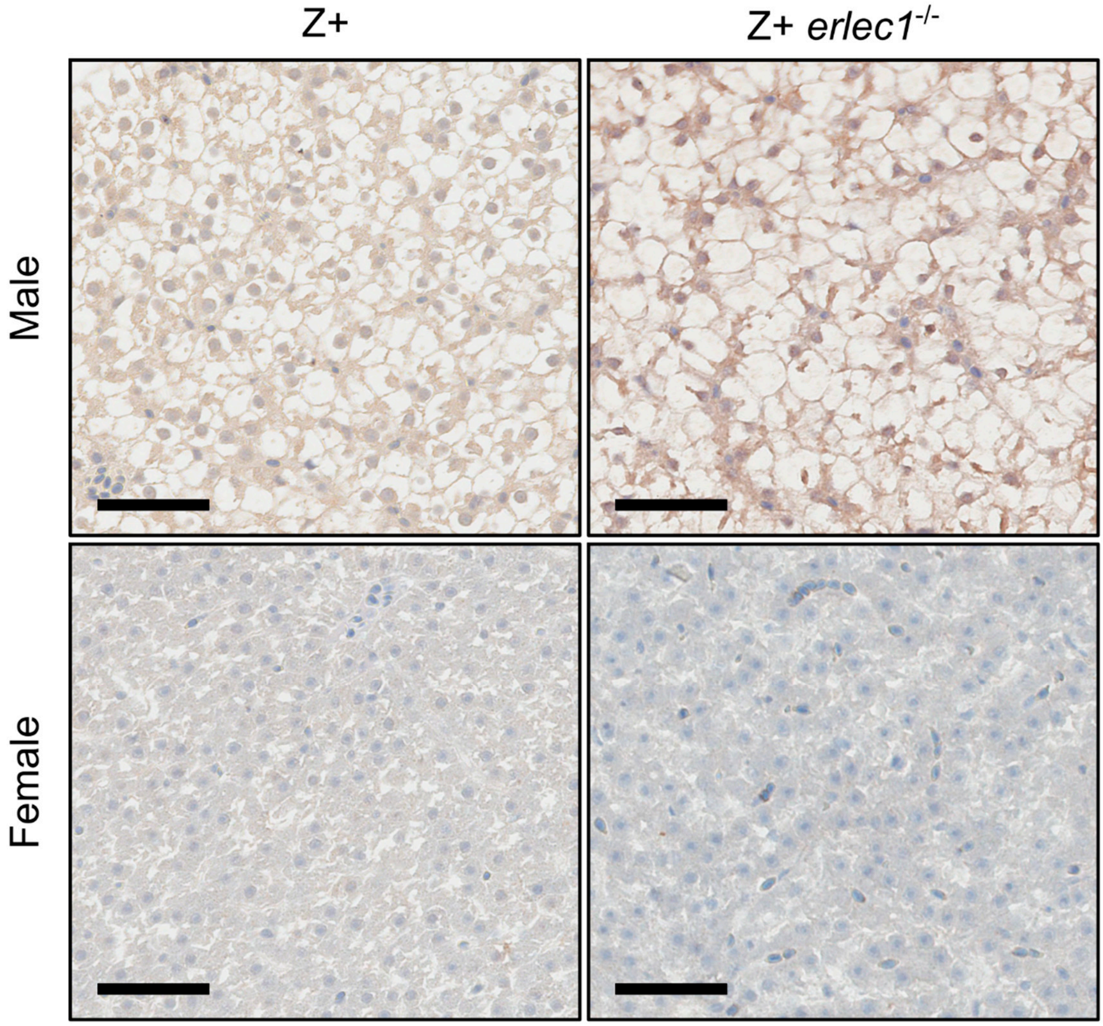

Figure S9. Immunohistochemical staining of *erlec1* mutant expressing ZAAT.
Representative images of 3 μm thick paraffin zebrafish liver sections stained for human antitrypsin. Scale bar represents 50 μm.

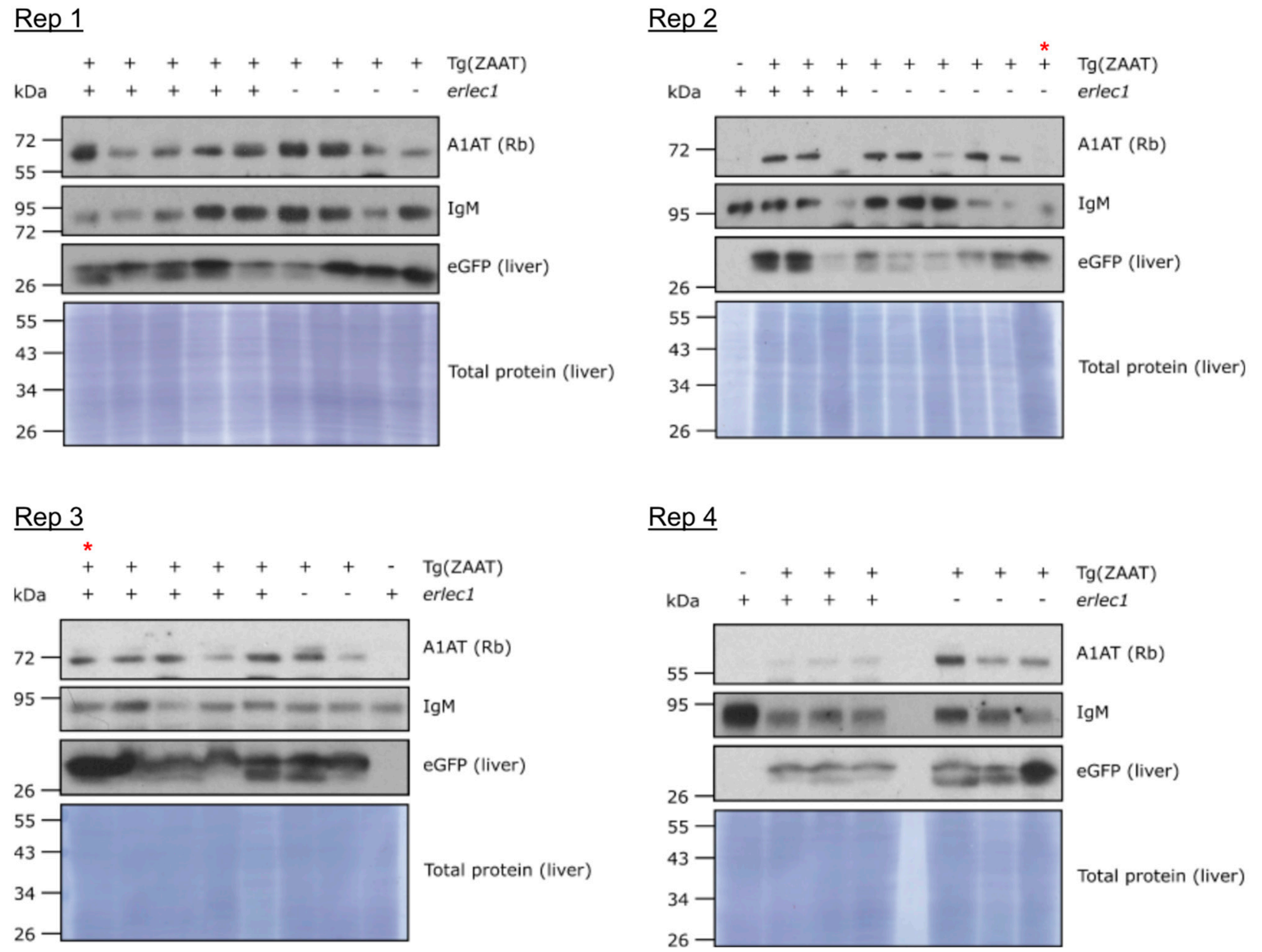

Figure S10. Immunoblots indicating the circulating levels of ZAAT in *erlec1*$^{-/-}$ zebrafish.
Red asterisks indicate outliers determined by the ROUT method (Q=1%), and these data are excluded from the analysis.

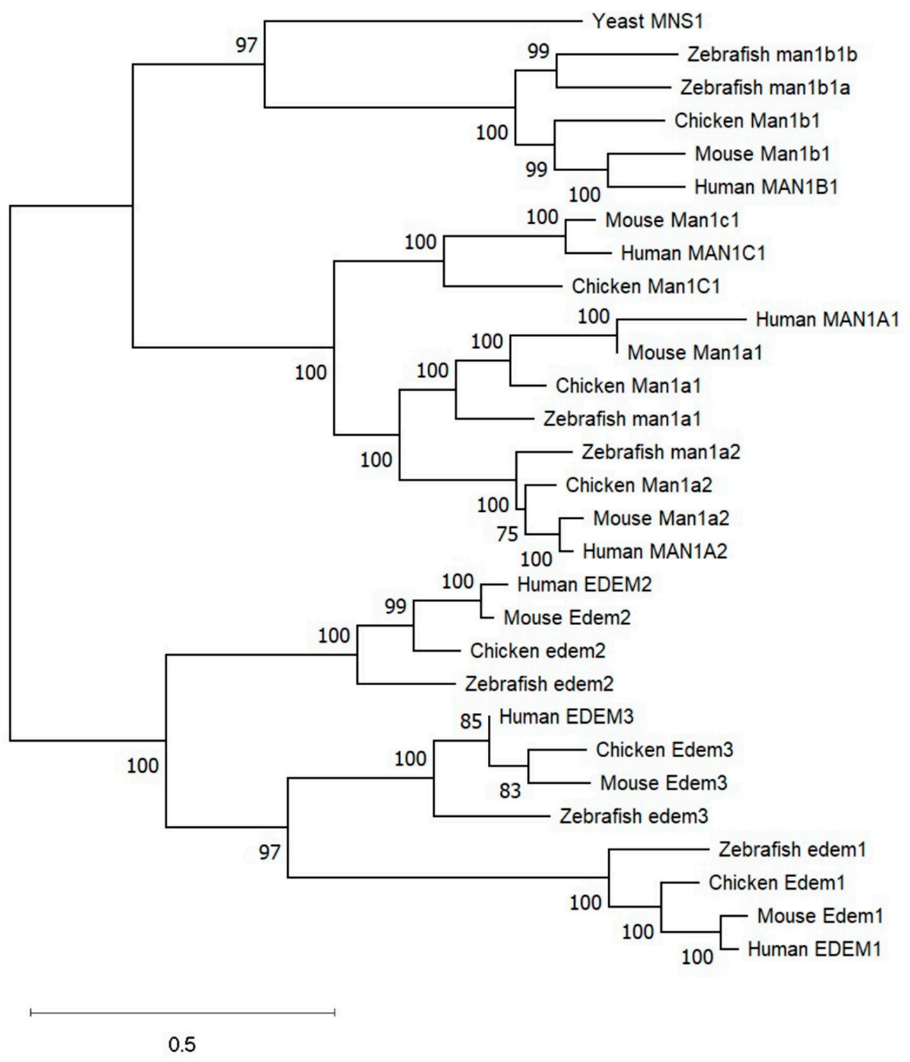

Figure S11. Phylogenetic analysis of glycoside hydrolase family 47 proteins.

Glycoside hydrolase family 47 (GH47) protein sequences from human, mouse, chicken, zebrafish, and yeast were aligned using Clustal-W. Evolutionary history was inferred by using the Maximum Likelihood method and Jones *et al*. w/freq. model. The tree was calculated from the alignment using Molecular Evolutionary Genetics Analysis software, version 10 (MEGA X). The percentage of trees in which the associated taxa clustered together in the bootstrap test (1000 replicates) are shown next to the branches. Initial tree(s) for the heuristic search were obtained automatically by applying Neighbor-Join and BioNJ algorithms to a matrix of pairwise distances estimated using the Jones-Taylor-Thornton (JTT) model. Scale bar refers to an evolutionary distance (number of substitutions per site) of 0.5.

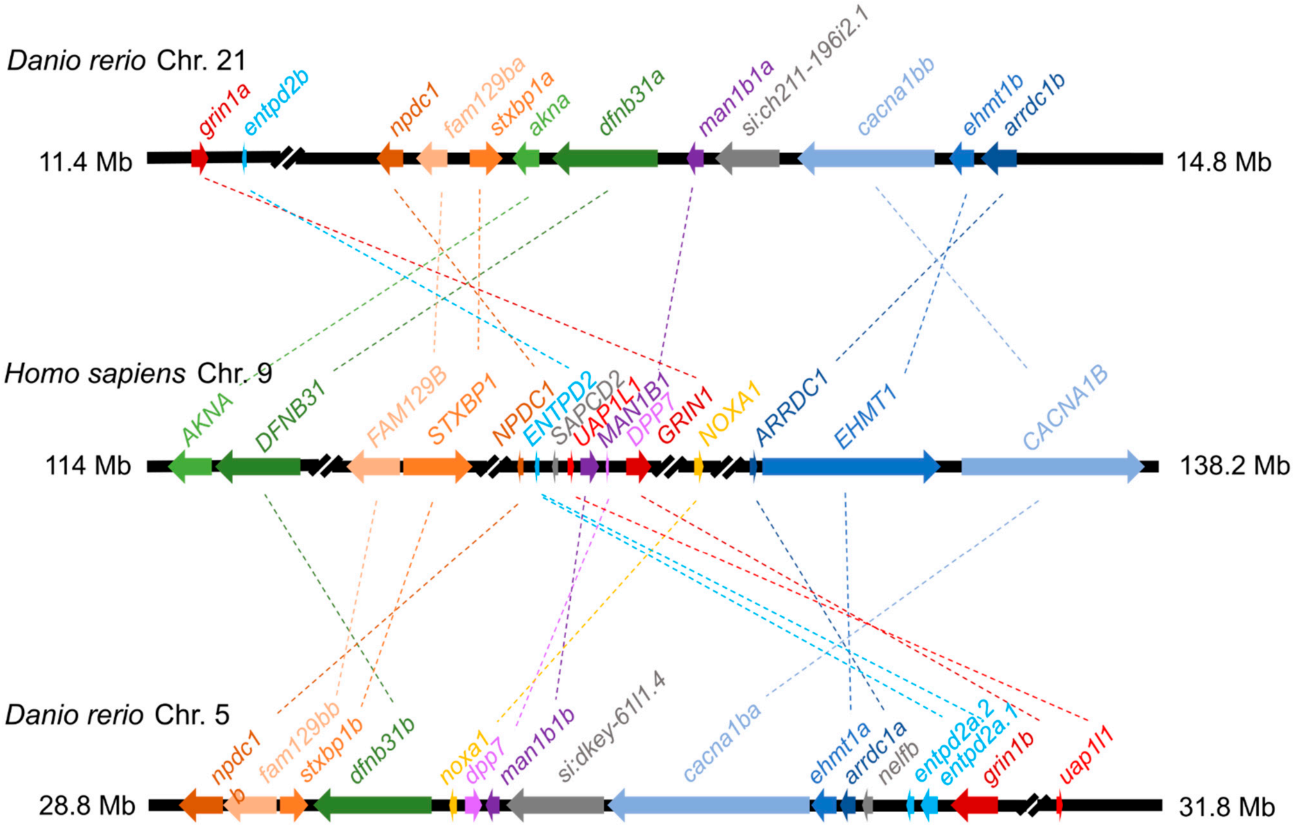

Figure S12. Synteny relationships of *man1b1* gene loci in human and zebrafish.
Mapped genomes of *Homo sapiens* and *Danio rerio* were used to identify conserved gene neighbours of *man1b1a* and *man1b1b*.

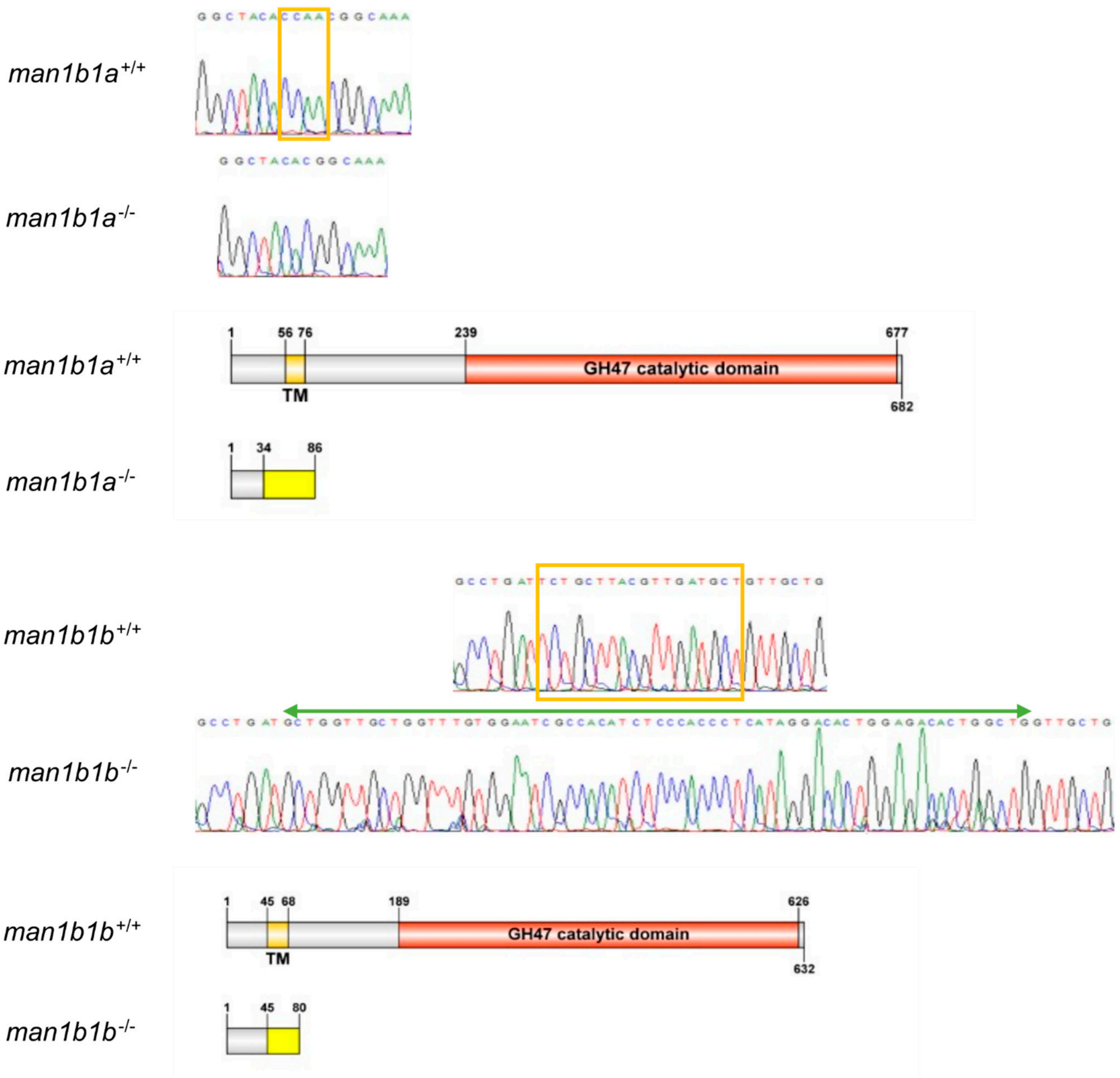

Figure S13. Sequence validation of *man1b1a* and *man1b1b* mutants.
Sequencing of F2 homozygotes confirms a 4 bp deletion at positions 101-104 for *man1b1a*, resulting in frameshift leading to insertion of 52 amino acids (yellow) at position 34 followed by a premature stop codon. Sequencing of *man1b1b* screening PCR product confirms an 18 bp deletion (outlined in yellow) with 65 bp insertion (indicated by green arrow), resulting in a net insertion of 47 bp and a frameshift leading to insertion of 35 amino acids (yellow) at position 45 followed by a premature stop codon. TM = transmembrane domain.

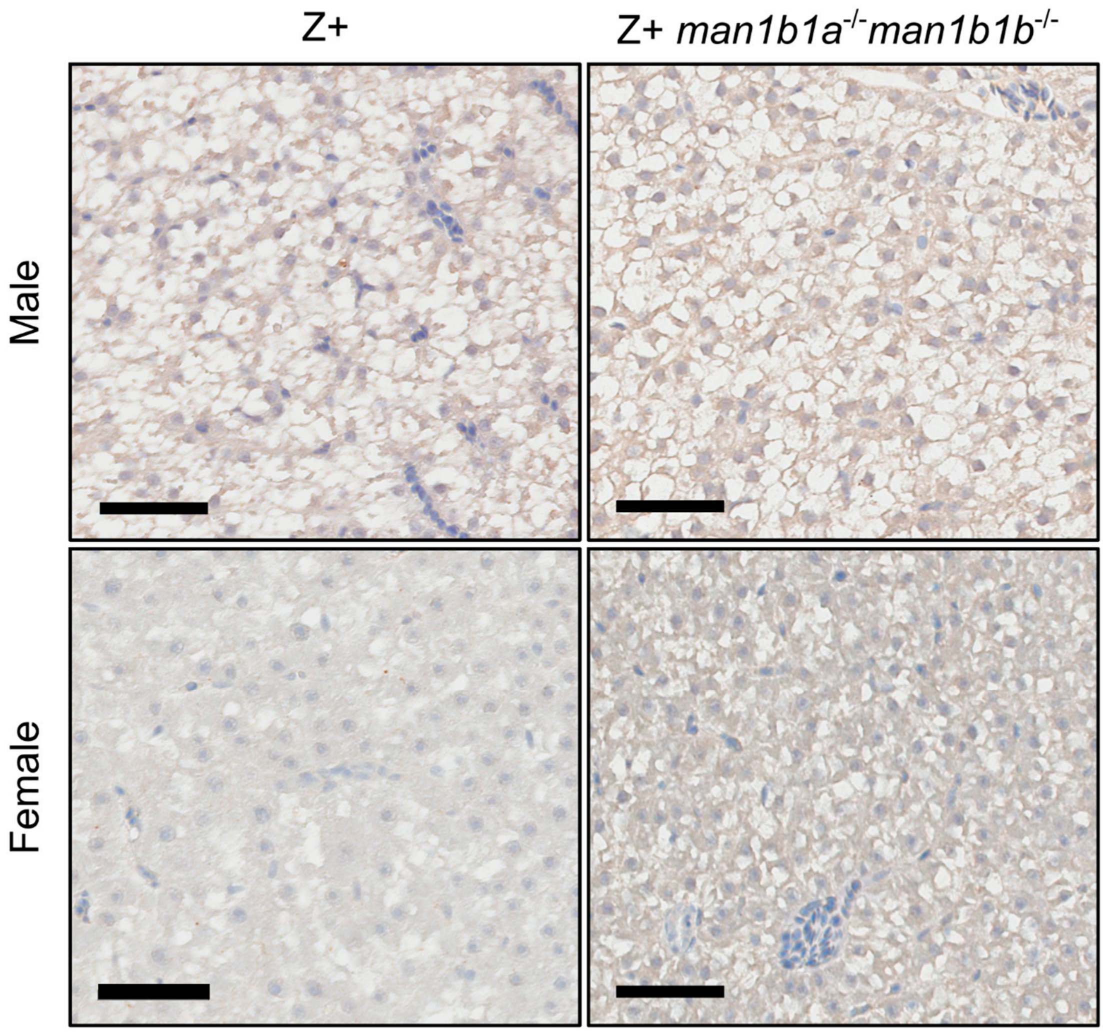

Figure S14. Immunohistochemical staining of *man1b1* double mutant expressing ZAAT. Representative images of 3 μm thick paraffin zebrafish liver sections stained for human antitrypsin. Scale bar represents 50 μm.

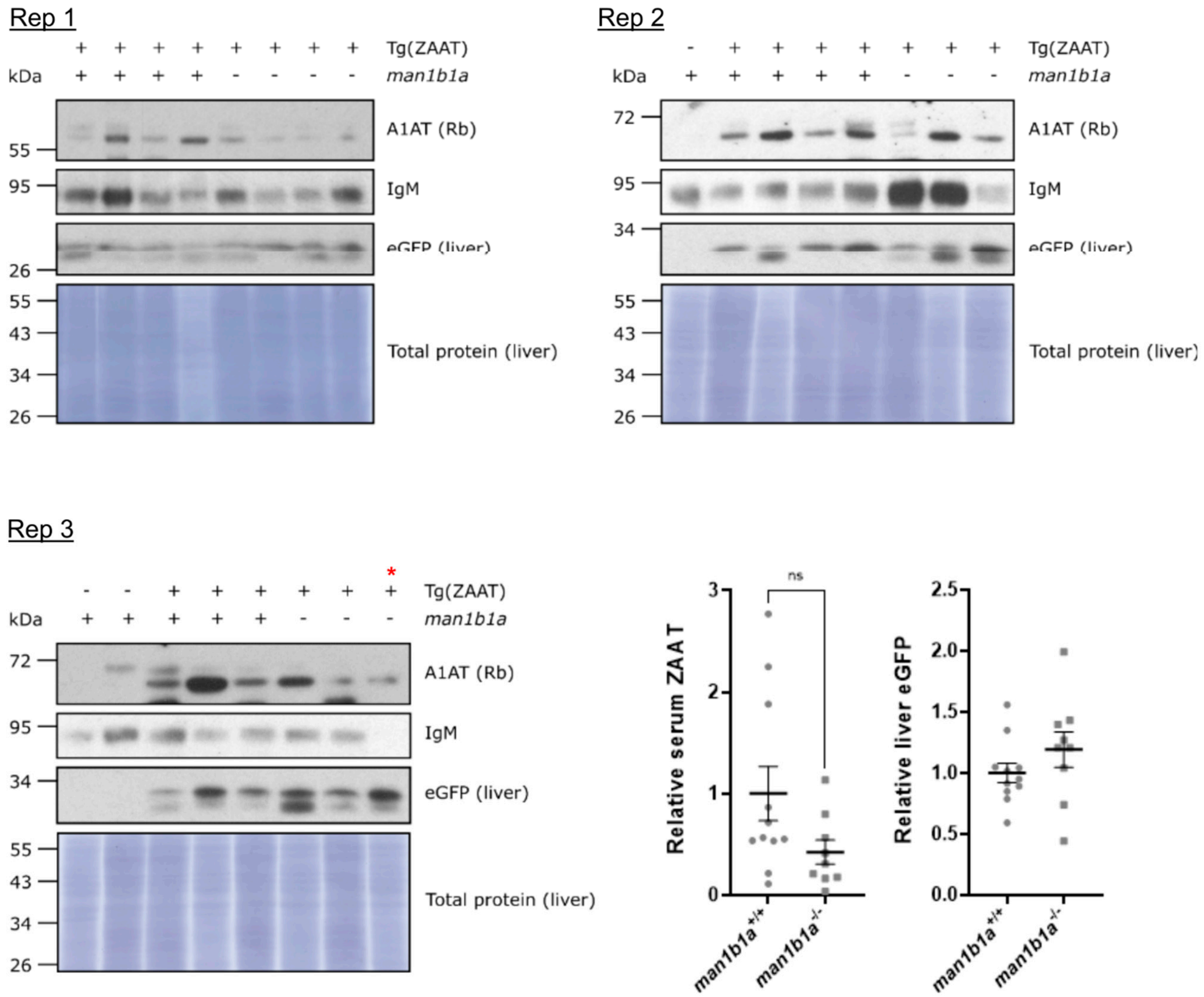

Figure S15. Immunoblots indicating the circulating levels of ZAAT in *man1b1a*$^{-/-}$ zebrafish. Red asterisks indicate outliers determined by the ROUT method (Q=1%), and these data are excluded from the analysis. Bottom right panel shows quantitation of protein signal by densitometry pooled from three independent experiments, with each point representing an individual fish. Statistical significance was determined by Unpaired Student's t-test. ns = non-significant.

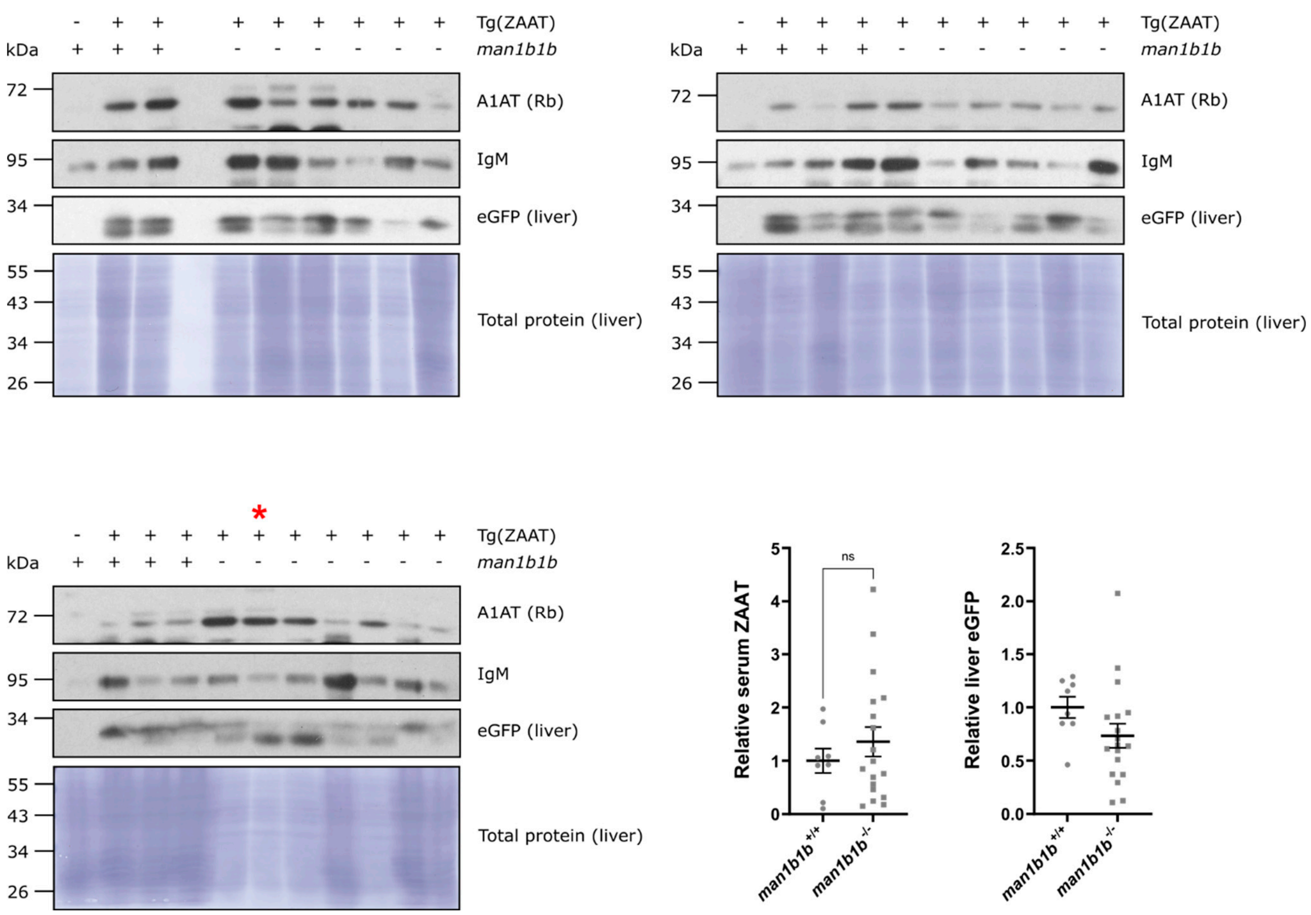

Figure S16. Immunoblots indicating the circulating levels of ZAAT in *man1b1b*$^{-/-}$ zebrafish

Red asterisks indicate outliers determined by the ROUT method (Q=1%), and these data are excluded from the analysis. Bottom right panel shows quantitation of protein signal by densitometry pooled from three independent experiments, with each point representing an individual fish. Statistical significance was determined by Unpaired Student's t-test. ns = non-significant.

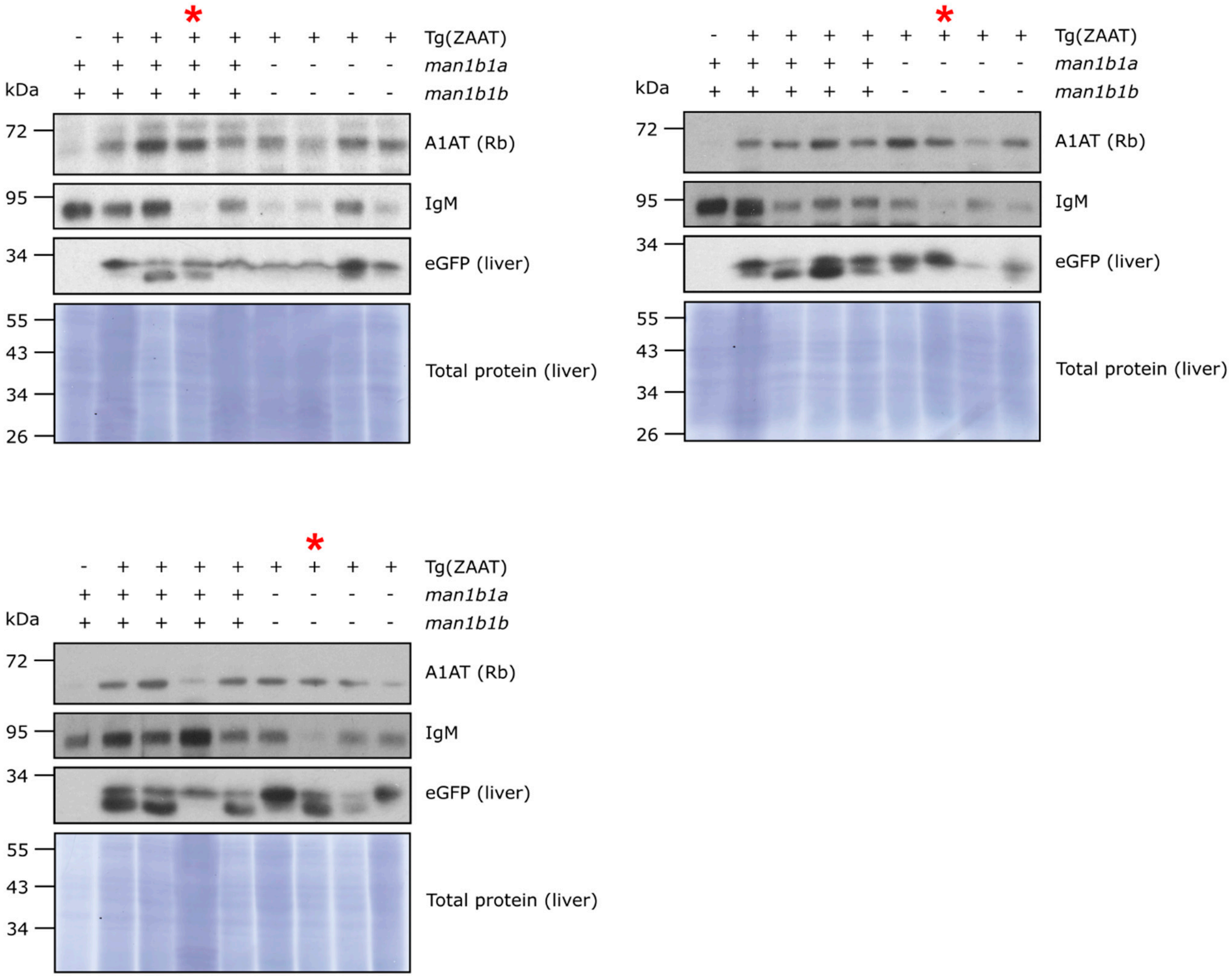

Figure S17. Immunoblots indicating the circulating levels of ZAAT in *man1b1a*$^{-/-}$ *man1b1b*$^{-/-}$ zebrafish.

Red asterisks indicate outliers determined by the ROUT method (Q=1%), and these data are excluded from the analysis.

Table S1. List of DEGs identified from RNA-seq

Gene	Product	Z+/A+	A+/A-	Z+/Z-
klhl24b	kelch-like family member 24b	16.89	0.86	12.13
frmd4bb	FERM domain containing 4Bb	16.35	0.56	8.00
bco2l	beta-carotene 15, 15-dioxygenase 2, like	8.13	1.72	5.86
fam222a	family with sequence similarity 222, member A	6.24	0.60	4.89
ulk2	unc-51 like autophagy activating kinase 2	6.17	1.25	6.45
lmbr1l	limb development membrane protein 1-like	6.16	0.53	2.23
fbxo25	F-box protein 25	6.05	1.77	9.06
si:ch211-232d10.1	si:ch211-232d10.1	5.73	0.91	3.36
exo5	exonuclease 5	5.4	1.00	5.10
ulk1a	unc-51 like autophagy activating kinase 1a	5.27	0.95	5.90
cyhr1	cysteine/histidine-rich 1	5.23	0.51	2.68
tle3b	transducin-like enhancer of split 3b	5.13	1.05	8.28
mtx3	metaxin 3	5.11	1.32	4.76
trim44	tripartite motif containing 44	4.95	0.85	3.46
si:ch73-386h18.1	si:ch73-386h18.1	4.95	0.80	4.20
irs2a	insulin receptor substrate 2a	4.84	0.86	3.51
klhl32	kelch-like family member 32	4.52	1.55	4.82
crbn	cereblon	4.37	1.02	3.23
stk11ip	serine/threonine kinase 11 interacting protein	4.34	0.68	5.66
peli2	pellino E3 ubiquitin protein ligase family member 2	4.21	0.66	3.68
slc34a2b	solute carrier family 34 (type II sodium/phosphate cotransporter), member 2b	4.14	0.84	3.58
abo	ABO blood group (transferase A, alpha 1-3-N-acetylgalactosaminyltransferase; transferase B, alpha 1-3-galactosyltransferase)	4.08	1.68	6.87
tesk2	testis-specific kinase 2	4.08	0.61	3.29
invs	inversin	4.04	0.95	2.69
ftr67	finTRIM family, member 67	4.02	0.89	3.41
nav2b	neuron navigator 2b	3.99	1.09	4.38
sik2a	salt-inducible kinase 2a	3.96	1.75	6.32
tbcela	tubulin folding cofactor E-like a	3.95	1.10	4.03
rapgef3	Rap guanine nucleotide exchange factor (GEF) 3	3.9	0.49	3.12
cyp2r1	cytochrome P450, family 2, subfamily R, polypeptide 1	3.83	1.11	4.23
nr2c2	nuclear receptor subfamily 2, group C, member 2	3.66	0.76	2.87
cylda	cylindromatosis (turban tumor syndrome), a	3.65	0.66	1.97
nocta	nocturnin a	3.62	0.95	2.46
hfe2	hemochromatosis type 2	3.59	0.74	3.56
lmbrd2b	LMBR1 domain containing 2b	3.56	0.69	2.17
ankib1b	ankyrin repeat and IBR domain containing 1b	3.52	0.94	3.51
rmdn2	regulator of microtubule dynamics 2	3.5	1.70	5.10
katnal1	katanin p60 subunit A-like 1	3.49	0.90	2.33
mtmr3	myotubularin related protein 3	3.49	1.21	3.84
evi5b	ecotropic viral integration site 5b	3.47	0.90	4.26
dcun1d4	DCN1, defective in cullin neddylation 1, domain containing 4 (S. cerevisiae)	3.44	1.01	4.44
tada2a	transcriptional adaptor 2A	3.34	0.91	2.89

bnip4	BCL2 interacting protein 4	3.33	0.79	2.41
pomk	protein-O-mannose kinase	3.29	0.88	2.79
wu:fb44b02	wu:fb44b02	3.23	1.21	2.99
wdr26a	WD repeat domain 26a	3.21	0.80	2.66
ssfa2	sperm specific antigen 2	3.12	0.73	1.80
kifap3a	kinesin-associated protein 3a	3.08	0.87	2.00
syne3	spectrin repeat containing, nuclear envelope family member 3	3.07	1.27	3.76
FO834800.1		3.01	0.50	2.01
wdr24	WD repeat domain 24	3	1.31	3.73
fam222ba	family with sequence similarity 222, member Ba	2.97	0.96	3.84
si:dkey-38p12.3	si:dkey-38p12.3	2.95	0.90	2.77
map3k4	mitogen-activated protein kinase kinase kinase 4	2.94	1.05	3.05
creb3l2	cAMP responsive element binding protein 3-like 2	2.93	0.90	3.14
rufy2	RUN and FYVE domain containing 2	2.91	1.11	2.57
si:dkey-40c23.1	si:dkey-40c23.1	2.91	1.44	2.99
rassf3	Ras association (RalGDS/AF-6) domain family member 3	2.9	1.24	2.10
agtpbp1	ATP/GTP binding protein 1	2.88	0.77	1.88
mtmr11	myotubularin related protein 11	2.81	1.56	4.06
nr3c1	nuclear receptor subfamily 3, group C, member 1 (glucocorticoid receptor)	2.8	0.88	2.50
fam160b2	family with sequence similarity 160, member B2	2.79	1.01	2.73
cpeb3	cytoplasmic polyadenylation element binding protein 3	2.72	0.18	0.38
si:dkey-246g23.4	si:dkey-246g23.4	2.71	1.97	6.82
coq8a	coenzyme Q8A	2.71	0.87	2.25
scube2	signal peptide, CUB domain, EGF-like 2	2.7	1.22	2.73
ulk1b	unc-51 like autophagy activating kinase 1	2.69	1.56	3.86
si:dkey-249d8.1	si:dkey-249d8.1	2.69	0.88	1.97
stard3	StAR-related lipid transfer (START) domain containing 3	2.68	0.63	1.78
arhgef16	Rho guanine nucleotide exchange factor (GEF) 16	2.68	1.51	2.79
rab11bb	RAB11B, member RAS oncogene family, b	2.67	0.81	1.84
hdac6	histone deacetylase 6	2.67	0.85	1.97
stag2a	stromal antigen 2a	2.67	0.81	2.00
si:ch211-235o23.1	si:ch211-235o23.1	2.65	0.79	2.01
nlrc5	NLR family, CARD domain containing 5	2.65	0.90	2.46
fndc3a	fibronectin type III domain containing 3A	2.64	0.76	2.27
si:dkey-29p10.4	si:dkey-29p10.4	2.61	0.71	1.91
brip1	BRCA1 interacting protein C-terminal helicase 1	2.61	1.40	2.89
zfyve26	zinc finger, FYVE domain containing 26	2.59	0.80	2.31
gfod2	glucose-fructose oxidoreductase domain containing 2	2.58	1.03	3.25
lrrc45	leucine rich repeat containing 45	2.58	1.24	3.20
zbtb41	zinc finger and BTB domain containing 41	2.58	1.25	3.01
vrk2	vaccinia related kinase 2	2.57	0.84	1.78
irgf1	immunity-related GTPase family, f1	2.56	0.80	1.87
zgc:100832	zgc:100832	2.54	0.95	2.22
inpp4aa	inositol polyphosphate-4-phosphatase type I Aa	2.54	1.49	3.41
si:dkey-205i10.1	si:dkey-205i10.1	2.54	1.63	4.08
ghdc	GH3 domain containing	2.53	0.88	1.87
kif7	kinesin family member 7	2.52	1.24	2.64

irf2	interferon regulatory factor 2	2.52	0.95	2.55
senp7b	SUMO1/sentrin specific peptidase 7b	2.51	1.26	3.63
si:ch211-85n16.4	si:ch211-85n16.4	2.5	0.92	2.58
met	MET proto-oncogene, receptor tyrosine kinase	2.49	0.82	3.12
nfkbie	nuclear factor of kappa light polypeptide gene enhancer in B-cells inhibitor, epsilon	2.49	1.03	3.66
zranb1b	zinc finger, RAN-binding domain containing 1b	2.47	1.20	3.12
flcn	folliculin	2.47	0.79	1.71
unkl	unkempt family zinc finger-like	2.47	0.92	1.89
si:ch211-216l23.2	si:ch211-216l23.2	2.47	0.95	2.22
herc3	HECT and RLD domain containing E3 ubiquitin protein ligase 3	2.47	1.16	2.36
zdhhc9	zinc finger, DHHC-type containing 9	2.46	0.96	2.39
trmt44	tRNA methyltransferase 44 homolog	2.46	0.84	2.03
mertka	c-mer proto-oncogene tyrosine kinase a	2.45	1.13	2.89
kcng4b	potassium voltage-gated channel, subfamily G, member 4b	2.44	4.31	23.59
wdtc1	WD and tetratricopeptide repeats 1	2.43	0.69	1.67
uacaa	uveal autoantigen with coiled-coil domains and ankyrin repeats a	2.43	1.61	3.10
tfpt	TCF3 (E2A) fusion partner	2.43	1.07	1.96
efcab7	EF-hand calcium binding domain 7	2.43	1.48	3.43
notch3	notch 3	2.42	1.16	2.22
si:dkey-24l11.2	si:dkey-24l11.2	2.41	0.85	1.92
zgc:153151	zgc:153151	2.41	0.90	2.17
bckdk	branched chain ketoacid dehydrogenase kinase	2.4	0.67	1.97
marveld2a	MARVEL domain containing 2a	2.4	1.01	2.27
als2b	amyotrophic lateral sclerosis 2b (juvenile)	2.4	1.60	2.62
nelfcd	negative elongation factor complex member C/D	2.4	0.96	1.91
abcd4	ATP-binding cassette, sub-family D (ALD), member 4	2.39	0.98	1.99
pxna	paxillin a	2.37	1.65	2.97
ddx59	DEAD (Asp-Glu-Ala-Asp) box polypeptide 59	2.37	1.10	2.46
fbxo3	F-box protein 3	2.37	1.08	2.64
si:dkey-240n22.3	si:dkey-240n22.3	2.36	1.98	3.46
creb3l3l	cAMP responsive element binding protein 3-like 3 like	2.35	0.71	1.62
fbxl6	F-box and leucine-rich repeat protein 6	2.35	1.36	3.61
arhgap27	Rho GTPase activating protein 27	2.33	1.36	2.23
man2c1	mannosidase, alpha, class 2C, member 1	2.33	0.83	1.72
si:ch73-138n13.1	si:ch73-138n13.1	2.33	1.14	2.85
CU207301.3		2.33	1.07	1.91
krit1	KRIT1, ankyrin repeat containing	2.32	1.33	2.51
CLIP4	si:dkeyp-47f9.4	2.31	1.31	2.53
CU856539.2		2.29	1.22	2.16
spast	spastin	2.28	1.22	3.53
smc6	structural maintenance of chromosomes 6	2.27	1.11	2.60
si:ch73-95l15.5	si:ch73-95l15.5	2.27	0.73	1.79
dgcr6	DiGeorge syndrome critical region gene 6	2.26	0.90	1.99
tspeara	thrombospondin-type laminin G domain and EAR repeats a	2.26	1.92	4.69
tnrc6c1	trinucleotide repeat containing 6C1	2.26	1.02	3.43
mfsd4b	major facilitator superfamily domain containing 4B	2.25	0.85	1.93
wdsub1	WD repeat, sterile alpha motif and U-box domain containing 1	2.25	0.82	1.84

recql5	RecQ helicase-like 5	2.25	0.92	1.67
ehmt1a	euchromatic histone-lysine N-methyltransferase 1a	2.25	1.13	2.50
si:dkey-72l17.5	si:dkey-72l17.5	2.24	1.51	2.06
tmem131l	transmembrane 131 like	2.24	0.99	1.89
zgc:158366	zgc:158366	2.23	0.74	1.55
sirt6	sirtuin 6	2.22	1.05	2.33
ncoa3	nuclear receptor coactivator 3	2.21	0.88	2.03
kdm4b	lysine (K)-specific demethylase 4B	2.2	0.80	1.93
zgc:173581	zgc:173581	2.19	1.01	2.00
rfx1b	regulatory factor X, 1b (influences HLA class II expression)	2.19	1.13	2.17
BX005416.1		2.19	1.08	2.73
slain2	SLAIN motif family, member 2	2.19	1.50	2.81
phf2	PHD finger protein 2	2.19	1.18	2.00
kif3a	kinesin family member 3A	2.18	1.13	2.25
arhgef1b	Rho guanine nucleotide exchange factor (GEF) 1b	2.18	0.91	1.51
pcdh12	protocadherin 12	2.18	1.47	3.63
CABZ01021435.1		2.18	1.32	2.89
capn7	calpain 7	2.18	0.75	1.71
sumf1	sulfatase modifying factor 1	2.17	0.93	1.61
si:dkey-159n16.2	si:dkey-159n16.2	2.17	2.01	3.20
arhgap27l	Rho GTPase activating protein 27, like	2.16	1.35	3.58
ep300b	E1A binding protein p300 b	2.16	1.34	3.34
ap4e1	adaptor-related protein complex 4, epsilon 1 subunit	2.16	0.99	2.04
zgc:123105	zgc:123105	2.16	0.90	1.42
ncoa1	nuclear receptor coactivator 1	2.16	1.40	3.07
calcoco1a	calcium binding and coiled-coil domain 1a	2.15	1.28	3.16
rabgap1	RAB GTPase activating protein 1	2.15	1.63	2.66
nav2a	neuron navigator 2a	2.14	1.62	6.96
dbpa	D site albumin promoter binding protein a	2.14	0.95	2.11
ift172	intraflagellar transport 172	2.14	1.19	2.04
si:dkey-165a24.9	si:dkey-165a24.9	2.14	1.38	2.48
ptpn2a	protein tyrosine phosphatase, non-receptor type 2, a	2.13	0.78	1.77
CR388364.2		2.13	2.13	4.82
nt5dc2	5'-nucleotidase domain containing 2	2.13	0.77	1.57
tead1a	TEA domain family member 1a	2.12	1.44	3.53
trpm6	transient receptor potential cation channel, subfamily M, member 6	2.12	1.74	2.45
pld1a	phospholipase D1a	2.12	0.74	1.79
trpm2	transient receptor potential cation channel, subfamily M, member 2	2.12	1.91	3.27
si:dkey-240n22.6	si:dkey-240n22.6	2.12	1.00	2.04
rnf20	ring finger protein 20, E3 ubiquitin protein ligase	2.12	1.00	2.11
cetn4	centrin 4	2.11	0.92	1.80
tmem120a	transmembrane protein 120A	2.11	1.16	2.19
mgea5	meningioma expressed antigen 5 (hyaluronidase)	2.1	0.97	2.73
brdt	bromodomain, testis-specific	2.1	1.85	3.05
usp2a	ubiquitin specific peptidase 2a	2.1	1.17	2.60
mtmr8	myotubularin related protein 8	2.09	1.36	2.33
sh3gl3a	SH3-domain GRB2-like 3a	2.08	0.97	3.71
qser1	glutamine and serine rich 1	2.08	1.14	2.41

clk4a	CDC-like kinase 4a	2.07	1.78	3.07
CR759794.1		2.07	1.17	2.58
als2a	amyotrophic lateral sclerosis 2a (juvenile)	2.07	1.28	2.03
lims1	LIM and senescent cell antigen-like domains 1	2.06	0.87	2.04
rb1cc1	RB1-inducible coiled-coil 1	2.03	0.67	1.45
ppp2r3c	protein phosphatase 2, regulatory subunit B", gamma	2.03	0.86	1.47
gpatch11	G patch domain containing 11	2.03	0.74	1.41
kdm4c	lysine (K)-specific demethylase 4C	2.03	0.98	1.83
smchd1	structural maintenance of chromosomes flexible hinge domain containing 1	2.02	0.78	1.56
epg5	ectopic P-granules autophagy protein 5 homolog (C. elegans)	2.01	1.30	2.31
chd1l	chromodomain helicase DNA binding protein 1-like	2	1.15	1.96
prkag2b	protein kinase, AMP-activated, gamma 2 non-catalytic subunit b	1.99	0.82	2.03
tjp2a	tight junction protein 2a (zona occludens 2)	1.98	0.90	1.99
chfr	checkpoint with forkhead and ring finger domains, E3 ubiquitin protein ligase	1.97	0.93	1.58
appl1	adaptor protein, phosphotyrosine interaction, PH domain and leucine zipper containing 1	1.97	0.84	1.88
nhsl1b	NHS-like 1b	1.97	1.48	2.73
zgc:153012	zgc:153012	1.96	0.88	2.04
si:dkey-262g12.14	si:dkey-262g12.14	1.95	1.40	2.35
actb2	actin, beta 2	1.95	0.83	1.64
nr1h3	nuclear receptor subfamily 1, group H, member 3	1.94	0.78	1.71
kmt2e	lysine (K)-specific methyltransferase 2E	1.94	1.24	2.57
zgc:152774	zgc:152774	1.93	1.03	1.87
shc2	SHC (Src homology 2 domain containing) transforming protein 2	1.93	1.07	2.45
def6c	differentially expressed in FDCP 6c homolog	1.92	0.85	2.38
mkrn1	makorin, ring finger protein, 1	1.92	1.20	2.71
pak2a	p21 protein (Cdc42/Rac)-activated kinase 2a	1.92	0.88	1.66
taf3	TAF3 RNA polymerase II, TATA box binding protein (TBP)-associated facto	1.92	0.88	1.78
raf1a	Raf-1 proto-oncogene, serine/threonine kinase a	1.91	1.04	1.82
selenoo1	selenoprotein O1	1.91	1.05	1.96
lnx2b	ligand of numb-protein X 2b	1.89	1.37	2.08
mcf2la	mcf.2 cell line derived transforming sequence-like a	1.89	0.96	1.68
cyldl	cylindromatosis (turban tumor syndrome), like	1.89	0.78	1.46
ptpn13	protein tyrosine phosphatase, non-receptor type 13	1.89	1.38	2.46
rbm18	RNA binding motif protein 18	1.89	0.88	1.84
alkbh3	alkB homolog 3, alpha-ketoglutarate-dependent dioxygenase	1.88	1.42	2.60
gnpnat1	glucosamine-phosphate N-acetyltransferase 1	1.88	1.15	2.79
ldb2a	LIM domain binding 2a	1.88	1.10	1.99
CZQB01141835.2		1.88	1.10	2.33
rab40b	RAB40B, member RAS oncogene family	1.87	0.79	1.77
dffb	DNA fragmentation factor, beta polypeptide (caspase-activated DNase)	1.87	0.97	1.99
dyrk1b	dual-specificity tyrosine-(Y)-phosphorylation regulated kinase 1B	1.87	1.05	2.36
per2	period circadian clock 2	1.85	1.09	2.30
gtf2a1l	general transcription factor IIA, 1-like	1.85	1.02	1.73
SLC35G1	si:ch211-197g18.2	1.85	1.04	1.79
wdr33	WD repeat domain 33	1.84	1.19	2.06
si:ch211-261p9.4	si:ch211-261p9.4	1.84	0.84	1.53
pkp3b	plakophilin 3b	1.84	0.90	1.85

usp48	ubiquitin specific peptidase 48	1.84	1.03	1.78
znf384l	zinc finger protein 384 like	1.84	1.24	2.20
fpgs	folylpolyglutamate synthase	1.84	1.06	1.56
ift52	intraflagellar transport 52 homolog (Chlamydomonas)	1.83	1.10	1.88
ormdl1	ORMDL sphingolipid biosynthesis regulator 1	1.83	1.17	2.31
arhgef11	Rho guanine nucleotide exchange factor (GEF) 11	1.83	1.32	2.03
si:ch211-213a13.5	si:ch211-213a13.5	1.83	2.08	2.69
safb	scaffold attachment factor B	1.82	1.15	1.74
znf451	zinc finger protein 451	1.82	0.87	1.53
pnpla7a	patatin-like phospholipase domain containing 7a	1.81	0.99	1.66
rreb1b	ras responsive element binding protein 1b	1.81	1.10	1.92
uhmk1	U2AF homology motif (UHM) kinase 1	1.8	1.69	2.38
znf438	zinc finger protein 438	1.8	1.94	3.36
mbtps2	membrane-bound transcription factor peptidase, site 2	1.79	0.96	1.79
wdr45	WD repeat domain 45	1.79	0.97	1.42
cnot6a	CCR4-NOT transcription complex, subunit 6a	1.79	1.08	2.00
shroom2a	shroom family member 2a	1.79	1.37	3.14
mtmr14	myotubularin related protein 14	1.79	0.84	1.52
ppp1r12c	protein phosphatase 1, regulatory subunit 12C	1.78	1.37	1.93
mtmr9	myotubularin related protein 9	1.77	0.99	1.51
cep290	centrosomal protein 290	1.77	1.35	2.22
wdr41	WD repeat domain 41	1.77	1.43	2.04
pi4k2b	phosphatidylinositol 4-kinase type 2 beta	1.75	0.98	1.65
dopey1	dopey family member 1	1.75	1.19	2.30
wipf2a	WAS/WASL interacting protein family, member 2a	1.75	0.93	1.66
pank2	pantothenate kinase 2	1.74	1.22	1.77
ankrd12	ankyrin repeat domain 12	1.74	1.25	2.06
tab1	TGF-beta activated kinase 1/MAP3K7 binding protein 1	1.74	0.96	1.53
abca3b	ATP-binding cassette, sub-family A (ABC1), member 3b	1.73	0.98	1.69
spg11	spastic paraplegia 11	1.73	1.16	1.85
cdc14b	cell division cycle 14B	1.73	1.68	2.48
si:dkey-97a13.6	si:dkey-97a13.6	1.72	1.04	2.07
carf	calcium responsive transcription factor	1.72	1.49	1.96
ssbp4	single stranded DNA binding protein 4	1.71	1.49	3.25
si:dkey-28a3.2	si:dkey-28a3.2	1.7	0.99	1.73
si:ch211-230g15.5	si:ch211-230g15.5	1.69	1.27	2.27
malt3	MALT paracaspase 3	1.69	1.04	2.50
im:7160594	im:7160594	1.69	0.84	1.54
fmnl3	formin-like 3	1.68	1.19	2.03
pi4kb	phosphatidylinositol 4-kinase, catalytic, beta	1.66	0.95	1.80
stag1a	stromal antigen 1a	1.66	1.68	2.41
csnk1db	casein kinase 1, delta b	1.65	1.27	1.82
dtnbp1a	dystrobrevin binding protein 1a	1.65	1.00	1.47
taf2	TAF2 RNA polymerase II, TATA box binding protein (TBP)-associated factor	1.65	1.06	1.57
ncoa5	nuclear receptor coactivator 5	1.65	1.05	1.83

usp25	ubiquitin specific peptidase 25	1.65	1.16	1.69
foxp1b	forkhead box P1b	1.64	1.25	1.93
nrbp2b	nuclear receptor binding protein 2b	1.64	1.49	2.23
ypel5	yippee-like 5	1.63	1.14	1.42
ftr79	finTRIM family, member 79	1.62	1.41	2.33
gpc4	glypican 4	1.62	1.07	1.74
si:dkey-156n14.5	si:dkey-156n14.5	1.62	1.26	1.95
gatsl2	GATS protein-like 2	1.62	1.34	2.06
phrf1	PHD and ring finger domains 1	1.62	1.03	1.88
trafd1	TRAF-type zinc finger domain containing 1	1.61	0.99	1.84
ctdsp1	CTD (carboxy-terminal domain, RNA polymerase II, polypeptide A) small phosphatase 1	1.61	0.94	1.51
atmin	ATM interactor	1.61	1.46	2.33
gnal	guanine nucleotide binding protein (G protein), alpha activating activity polypeptide, olfactory type	1.6	1.51	2.38
tjp1a	tight junction protein 1a	1.6	1.25	2.16
si:dkey-16p6.1	si:dkey-16p6.1	1.6	1.26	1.97
wdr26b	WD repeat domain 26b	1.6	0.81	1.54
rbm4.2	RNA binding motif protein 4.2	1.58	1.06	1.55
golga4	golgin A4	1.58	1.01	1.47
rest	RE1-silencing transcription factor	1.57	1.98	3.29
yy1a	YY1 transcription factor a	1.57	1.01	1.52
slkb	STE20-like kinase b	1.57	1.55	2.30
FBXO48	si:dkey-202b17.4	1.56	1.12	1.40
grhpra	glyoxylate reductase/hydroxypyruvate reductase a	1.56	1.45	2.00
traf2b	Tnf receptor-associated factor 2b	1.56	1.13	1.64
si:ch73-1a9.3	si:ch73-1a9.3	1.54	1.19	1.60
brd9	bromodomain containing 9	1.53	1.01	1.44
inpp5ka	inositol polyphosphate-5-phosphatase Ka	1.5	0.97	1.38
add3a	adducin 3 (gamma) a	1.5	1.15	1.57
hook2	hook microtubule-tethering protein 2	1.49	0.99	1.36
per3	period circadian clock 3	1.48	1.30	1.73
tial1	TIA1 cytotoxic granule-associated RNA binding protein-like 1	1.47	1.43	1.88
hnrnpm	heterogeneous nuclear ribonucleoprotein M	1.47	1.07	1.55
usp8	ubiquitin specific peptidase 8	1.45	0.89	1.38
dusp16	dual specificity phosphatase 16	1.43	1.08	1.40
optn	optineurin	1.4	0.46	0.62
psmc1a	proteasome 26S subunit, ATPase 1a	1.36	0.53	0.67
arid4a	AT rich interactive domain 4A (RBP1-like)	1.36	1.20	1.52
cdh1	cadherin 1, type 1, E-cadherin (epithelial)	1.36	1.12	1.51
tfb2m	transcription factor B2, mitochondrial	0.76	1.00	0.70
upf1	upf1 regulator of nonsense transcripts homolog (yeast)	0.72	0.92	0.70
tomm22	translocase of outer mitochondrial membrane 22 homolog (yeast)	0.72	0.88	0.60
uba3	ubiquitin-like modifier activating enzyme 3	0.71	0.82	0.55
rbm25b	RNA binding motif protein 25b	0.71	0.95	0.74
srsf5a	serine/arginine-rich splicing factor 5a	0.7	0.83	0.63
sestd1	SEC14 and spectrin domains 1	0.7	0.73	0.53
mef2cb	myocyte enhancer factor 2cb	0.68	0.87	0.69

azin1a	antizyme inhibitor 1a	0.68	0.86	0.57
oaz1b	ornithine decarboxylase antizyme 1b	0.68	0.80	0.59
hmgb3b	high mobility group box 3b	0.67	0.98	0.64
si:dkey-286j15.1	si:dkey-286j15.1	0.65	0.98	0.63
lcn15	lipocalin 15	0.65	1.13	0.70
mapkapk2a	mitogen-activated protein kinase-activated protein kinase 2a	0.65	0.95	0.54
tomm70a	translocase of outer mitochondrial membrane 70 homolog A (S. cerevisiae)	0.65	0.94	0.59
cux2b	cut-like homeobox 2b	0.64	0.81	0.48
smim12	small integral membrane protein 12	0.63	0.75	0.48
rab35b	RAB35, member RAS oncogene family b	0.63	0.94	0.60
adck5	aarF domain containing kinase 5	0.62	0.57	0.34
nde1	nudE neurodevelopment protein 1	0.62	0.96	0.54
rap1ab	RAP1A, member of RAS oncogene family b	0.62	0.94	0.55
ddx61	DEAD (Asp-Glu-Ala-Asp) box helicase 61	0.62	0.90	0.55
znf574	zinc finger protein 574	0.62	0.76	0.55
kat2a	K(lysine) acetyltransferase 2A	0.61	0.82	0.55
ddx19	DEAD/H (Asp-Glu-Ala-Asp/His) box polypeptide 19 (DBP5 homolog, yeast)	0.61	0.96	0.53
dolpp1	dolichyldiphosphatase 1	0.61	0.91	0.61
si:ch211-210c8.7	si:ch211-210c8.7	0.61	1.16	0.65
mt-nd6	NADH dehydrogenase 6, mitochondrial	0.61	1.11	0.63
timm10b	translocase of inner mitochondrial membrane 10 homolog B (yeast)	0.6	1.06	0.64
kdm6al	lysine (K)-specific demethylase 6A, like	0.6	0.92	0.66
mapre1b	microtubule-associated protein, RP/EB family, member 1b	0.6	0.92	0.52
lsm1	LSM1, U6 small nuclear RNA associated	0.6	1.13	0.66
slc51a	solute carrier family 51, alpha subunit	0.6	0.96	0.53
pgam1a	phosphoglycerate mutase 1a	0.59	1.03	0.62
si:ch211-235e9.8	si:ch211-235e9.8	0.59	0.82	0.45
ppa1b	pyrophosphatase (inorganic) 1b	0.59	1.00	0.58
pinx1	PIN2/TERF1 interacting, telomerase inhibitor 1	0.58	0.95	0.52
fam136a	family with sequence similarity 136, member A	0.58	0.73	0.38
phospho2	phosphatase, orphan 2	0.58	1.05	0.59
mars	methionyl-tRNA synthetase	0.57	0.86	0.49
hmbsa	hydroxymethylbilane synthase a	0.56	0.95	0.48
si:dkey-121a9.3	si:dkey-121a9.3	0.56	0.92	0.49
qars	glutaminyl-tRNA synthetase	0.56	0.82	0.46
igdcc4	immunoglobulin superfamily, DCC subclass, member 4	0.56	0.67	0.46
ptpn11b	protein tyrosine phosphatase, non-receptor type 11, b	0.56	0.77	0.51
higd1a	HIG1 hypoxia inducible domain family, member 1A	0.56	1.04	0.56
si:ch73-21k16.5	si:ch73-21k16.5	0.56	1.06	0.54
elavl1a	ELAV like RNA binding protein 1a	0.55	1.06	0.55
ppih	peptidylprolyl isomerase H (cyclophilin H)	0.54	0.86	0.45
tor3a	torsin family 3, member A	0.54	0.93	0.55
hprt1	hypoxanthine phosphoribosyltransferase 1	0.54	0.90	0.48
sigmar1	sigma non-opioid intracellular receptor 1	0.54	0.94	0.50
cers2b	ceramide synthase 2b	0.54	0.79	0.40
si:ch211-51e12.7	si:ch211-51e12.7	0.53	1.22	0.69
nsdhl	NAD(P) dependent steroid dehydrogenase-like	0.53	1.03	0.52
btaf1	BTAF1 RNA polymerase II, B-TFIID transcription factor-associated	0.53	1.25	0.70

lgalsla	lectin, galactoside-binding-like a	0.53	1.06	0.53
foxo3a	forkhead box O3A	0.53	0.65	0.40
CDPF1	si:ch211-239e6.4	0.52	1.06	0.49
upp1	uridine phosphorylase 1	0.52	0.76	0.53
nt5dc3	5'-nucleotidase domain containing 3	0.52	0.94	0.51
pus10	pseudouridylate synthase 10	0.52	0.96	0.50
atp13a3	ATPase 13A3	0.51	0.47	0.23
ccnt2b	cyclin T2b	0.5	0.97	0.48
slc19a2	solute carrier family 19 (thiamine transporter), member 2	0.5	0.97	0.40
srd5a1	steroid-5-alpha-reductase, alpha polypeptide 1 (3-oxo-5 alpha-steroid delta 4-dehydrogenase alpha 1)	0.49	0.87	0.40
ABCF3	ATP binding cassette subfamily F member 3	0.49	0.75	0.38
znrd1	zinc ribbon domain containing 1	0.49	0.87	0.47
eif4e1c	eukaryotic translation initiation factor 4E family member 1c	0.49	1.01	0.48
cyb5b	cytochrome b5 type B	0.49	1.06	0.40
cyb5r2	cytochrome b5 reductase 2	0.48	0.86	0.38
grtp1a	growth hormone regulated TBC protein 1a	0.47	0.58	0.29
nudt3b	nudix (nucleoside diphosphate linked moiety X)-type motif 3b	0.47	0.74	0.39
znf1007	zinc finger protein 1007	0.47	0.93	0.55
sema4ba	sema domain, immunoglobulin domain (Ig), transmembrane domain (TM) and short cytoplasmic domain, (semaphorin) 4Ba	0.47	0.75	0.33
CABZ01002690.1		0.46	0.74	0.37
xpo4	exportin 4	0.45	1.16	0.43
rin2	Ras and Rab interactor 2	0.45	0.92	0.44
ptges3b	prostaglandin E synthase 3b (cytosolic)	0.44	1.07	0.43
myo10l3	myosin X-like 3	0.44	0.72	0.38
timm50	translocase of inner mitochondrial membrane 50 homolog (S. cerevisiae)	0.44	1.02	0.41
spag1a	sperm associated antigen 1a	0.44	0.58	0.27
gpam	glycerol-3-phosphate acyltransferase, mitochondrial	0.43	0.71	0.30
rpz2	rapunzel 2	0.43	1.11	0.49
asmt2	acetylserotonin O-methyltransferase 2	0.42	0.52	0.36
hs3st3b1b	heparan sulfate (glucosamine) 3-O-sulfotransferase 3B1b	0.42	0.84	0.50
arhgef2	rho/rac guanine nucleotide exchange factor (GEF) 2	0.4	0.87	0.28
pacs2	phosphofurin acidic cluster sorting protein 2	0.4	0.50	0.30
NC_002333.19		0.39	0.94	0.46
rbp7a	retinol binding protein 7a, cellular	0.39	0.54	0.18
frya	furry homolog a (Drosophila)	0.38	0.49	0.20
aacs	acetoacetyl-CoA synthetase	0.37	0.65	0.22
si:ch211-197l9.2	si:ch211-197l9.2	0.37	0.31	0.13
dhh	desert hedgehog	0.37	1.02	0.45
kirrel3l	kirre like nephrin family adhesion molecule 3, like	0.36	1.27	0.37
calcrlb	calcitonin receptor-like b	0.36	1.24	0.58
ptp4a3	protein tyrosine phosphatase type IVA, member 3	0.36	0.83	0.31
gtf3c2	general transcription factor IIIC, polypeptide 2, beta	0.35	0.94	0.43
dcbld1	discoidin, CUB and LCCL domain containing 1	0.34	0.38	0.13
slc38a3a	solute carrier family 38, member 3a	0.34	0.99	0.36
idi1	isopentenyl-diphosphate delta isomerase 1	0.34	1.17	0.31
si:ch1073-296i8.2	si:ch1073-296i8.2	0.33	0.83	0.15

CABZ01015475.1		0.33	1.09	0.41
rbms2b	RNA binding motif, single stranded interacting protein 2b	0.27	1.69	0.41
gramd4a	GRAM domain containing 4a	0.27	1.49	0.40
NC_002333.2		0.27	0.38	0.13
ebp	emopamil binding protein (sterol isomerase)	0.26	1.12	0.22
fdft1	farnesyl-diphosphate farnesyltransferase 1	0.26	1.25	0.27
hsd17b7	hydroxysteroid (17-beta) dehydrogenase 7	0.26	1.37	0.34
fgf13b	fibroblast growth factor 13b	0.25	0.52	0.12
mvda	mevalonate (diphospho) decarboxylase a	0.25	1.35	0.26
msmo1	methylsterol monooxygenase 1	0.23	1.89	0.31
sqlea	squalene epoxidase a	0.18	1.88	0.16
bach2a	BTB and CNC homology 1, basic leucine zipper transcription factor 2a	0.18	0.57	0.17
lss	lanosterol synthase (2,3-oxidosqualene-lanosterol cyclase)	0.17	1.19	0.14
mvk	mevalonate kinase	0.16	1.81	0.28
hmgcs1	3-hydroxy-3-methylglutaryl-CoA synthase 1 (soluble)	0.15	1.44	0.16
fdps	farnesyl diphosphate synthase (farnesyl pyrophosphate synthetase, dimethylallyltranstransferase, geranyltranstransferase)	0.14	2.39	0.27
igsf9ba	immunoglobulin superfamily, member 9Ba	0.12	1.71	0.33
rpp25a	ribonuclease P and MRP subunit p25, a	0.1	1.97	0.12
zbtb16a	zinc finger and BTB domain containing 16a	0.07	1.05	0.09

Table S2. List of DEPs identified from LC-MS/MS

Gene	Product	Z+/A+	A+/A-	Z+/Z-
Scgn	secretagogin, EF-hand calcium binding protein	9.89	1.18	2.76
Mgst2	microsomal glutathione S-transferase 2	5.18	0.52	3.52
GFP-T2A-AAT-transgene	GFP-T2A-AAT-transgene	4.63	1.02	5.59
Sdsl	serine dehydratase-like	3.43	2.98	1.93
Liph	lipase member H	3.13	0.70	5.05
Vmo1a	vitelline membrane outer layer 1 homolog a	2.77	1.52	1.95
Lrp1aa	low density lipoprotein receptor-related protein 1Aa	2.36	0.79	1.16
Si:ch211-74f19.2	si:ch211-74f19.2	1.95	0.71	2.42
Mpdu1b	mannose-P-dolichol utilization defect 1b	1.93	0.79	1.26
Fuk	fucokinase	1.65	0.86	1.09
Caska	calcium/calmodulin-dependent serine protein kinase a	1.64	0.79	1.52
Psmg1	proteasome (prosome, macropain) assembly chaperone 1	1.55	0.52	0.84
Sccpdhb	saccharopine dehydrogenase b	1.54	0.84	1.12
Larp4aa	La ribonucleoprotein domain family, member 4Aa	1.52	0.47	0.73
Mipep	mitochondrial intermediate peptidase	1.5	0.87	1.27
Anxa11b	annexin A11b	1.48	0.99	1.64
Sil1	SIL1 nucleotide exchange factor	1.45	0.48	0.86
Rrbp1b	ribosome binding protein 1b	1.43	0.23	0.55
Farsa	phenylalanyl-tRNA synthetase, alpha subunit	1.43	0.58	0.69
Zgc:152945	zgc:152945	1.41	0.40	0.73
Pcyt2	phosphate cytidylyltransferase 2, ethanolamine	1.4	0.53	0.76
Apobb.2	apolipoprotein Bb, tandem duplicate 2	1.38	0.88	1.41
Ipo7	importin 7	1.36	0.71	0.71
Setd1a	SET domain containing 1A	1.33	0.47	0.73
Erlec1	endoplasmic reticulum lectin 1	1.32	0.63	0.71
Dpm3	dolichyl-phosphate mannosyltransferase polypeptide 3	1.31	0.58	0.46
Larsa	leucyl-tRNA synthetase a	1.3	0.60	0.79
Rock2a	rho-associated, coiled-coil containing protein kinase 2a	1.29	0.85	1.43
Memo1	mediator of cell motility 1	1.29	0.74	0.83
Rab1ab	RAB1A, member RAS oncogene family b	1.27	0.62	0.84
Tmem177	transmembrane protein 177	1.27	0.97	1.31
Zgc:101540	zgc:101540	1.26	0.95	1.57
C8b	complement component 8, beta polypeptide	1.25	0.93	1.46
Fam3a	family with sequence similarity 3, member A	1.25	0.73	0.82
Abraxas2b	abraxas 2b, BRISC complex subunit	1.23	1.02	1.14
Sec61b	Sec61 translocon beta subunit	1.23	0.71	0.59
Vcp	valosin containing protein	1.22	0.67	0.74
Drg1	developmentally regulated GTP binding protein 1	1.21	0.70	0.81
Slc16a2	solute carrier family 16 member 2	1.2	0.80	0.78
Ufl1	UFM1-specific ligase 1	1.2	0.79	0.73
Gsptl1	G1 to S phase transition 1, like	1.19	0.70	0.76
Rab1aa	RAB1A, member RAS oncogene family a	1.19	0.70	0.85
Psma2	proteasome subunit alpha 2	1.17	0.80	0.85
Slc23a1	solute carrier family 23 (ascorbic acid transporter), member 1	1.15	1.00	1.15
Pafah1b1a	platelet-activating factor acetylhydrolase 1b, regulatory subunit 1a	1.13	0.81	0.87

Rab5c	RAB5C, member RAS oncogene family	1.12	0.80	0.94
Ufd1l	ubiquitin recognition factor in ER associated degradation 1	1.11	0.86	0.82
Psmd7	proteasome 26S subunit, non-ATPase 7	1.09	0.90	0.83
Mest	mesoderm specific transcript	0.9	1.06	0.93
Tmed7	transmembrane p24 trafficking protein 7	0.87	0.93	0.93
Cfl1	cofilin 1	0.86	1.03	0.88
Erp44	endoplasmic reticulum protein 44	0.84	0.95	0.85
Srsf5b	serine/arginine-rich splicing factor 5b	0.82	1.15	0.85
Enpp1	ectonucleotide pyrophosphatase/phosphodiesterase 1	0.82	1.55	1.58
Pyroxd2	pyridine nucleotide-disulphide oxidoreductase domain 2	0.82	1.05	0.90
Slc12a4	solute carrier family 12 (potassium/chloride transporter), member 4	0.81	1.06	0.79
Im:7138535	im:7138535	0.8	1.15	0.83
Dpy19l11	dpy-19-like 1, like (H. sapiens)	0.8	1.73	1.21
Cct2	haperonin containing TCP1 complex subunit 2	0.78	1.31	1.24
Gale	UDP-galactose-4-epimerase	0.76	1.23	0.81
Epcam	epithelial cell adhesion molecule	0.75	0.90	0.71
Snrpd3l	small nuclear ribonucleoprotein D3 polypeptide, like	0.74	0.93	0.61
Aldh9a1a	aldehyde dehydrogenase 9 family, member A1a	0.71	1.22	0.95
Rdh14b	retinol dehydrogenase 14b	0.69	1.17	0.84
Gpa33	glycoprotein A33 (transmembrane)	0.68	1.04	0.52
Mgc173689	MGC: 173689	0.63	0.93	0.78
Coro1a	coronin, actin binding protein, 1A	0.6	0.93	0.60
Krt91	keratin 91	0.57	1.20	0.70
Zgc:103586	zgc:103586	0.57	1.54	0.84
Tcea3	transcription elongation factor A (SII), 3	0.53	0.97	0.49
Ckmb	creatine kinase, muscle b	0.48	1.09	0.46
Nudt7	nudix (nucleoside diphosphate linked moiety X)-type motif 7	0.46	0.78	0.37
Zgc:174259	zgc:174259	0.38	0.87	0.45
Dap1b	death associated protein 1b	0.37	1.31	0.29
Pla2g1b	phospholipase A2, group IB (pancreas)	0.36	3.65	0.15
Pck1	phosphoenolpyruvate carboxykinase 1 (soluble)	0.35	0.72	0.18
Si:ch1073-110a20.3	si:ch1073-110a20.3	0.28	1.51	0.21

Table S3. GO enrichment from RNA-seq DEGs

	Gene Ontology Term (GO term)	Enrichment score
1	Response To Purine-Containing Compound	34.711
2	Isoprenoid Biosynthetic Process	30.819
3	Cholesterol Biosynthetic Process	30.639
4	Regulation Of Endosome Size	29.2
5	Autophagy Of Host Cells Involved In Interaction With Symbiont	27.769
6	Regulation Of Cholesterol Biosynthetic Process	25.244
7	Isopentenyl Diphosphate Biosynthetic Process, Mevalonate Pathway	19.467
8	Positive Regulation Of Rac Protein Signal Transduction	19.467
9	Positive Regulation Of Transcription From Rna Polymerase Ii Promoter Involved In Unfolded Protein Response	19.467
10	Glycerol-3-Phosphate Metabolic Process	19.467
11	Sterol Biosynthetic Process	16.686
12	Positive Regulation Of Protein Homodimerization Activity	15.427
13	Positive Regulation Of Hydrogen Peroxide-Mediated Programmed Cell Death	14.6
14	Negative Regulation Of Activation-Induced Cell Death Of T Cells	14.6
15	Regulation Of Autophagy	13.017
16	Receptor Recycling	12.622
17	Regulation Of Androgen Receptor Signaling Pathway	12.622
18	Cellular Response To Nerve Growth Factor Stimulus	12.24
19	Protein Localization	11.733
20	Dolichyl Diphosphate Biosynthetic Process	11.68
21	Positive Regulation Of Chromosome Segregation	11.68
22	Parkin-Mediated Stimulation Of Mitophagy In Response To Mitochondrial Depolarization	11.68
23	Regulation Of Establishment Of Protein Localization	11.68
24	Regulation Of Kinase Activity	11.68
25	Internal Peptidyl-Lysine Acetylation	11.68
26	Regulation Of Fatty Acid Metabolic Process	11.68
27	Liver Development	11.412
28	Phosphatidylinositol Biosynthetic Process	11.124
29	Positive Regulation Of Microtubule Polymerization	10.157
30	Regulation Of Rho Protein Signal Transduction	10.037
31	Peptidyl-Tyrosine Dephosphorylation	9.898
32	Optic Nerve Development	9.733
33	Regulation Of Phosphatidylinositol Dephosphorylation	9.733
34	Response To Fungicide	9.733
35	Regulation Of Tumor Necrosis Factor Production	9.733
36	Cytoplasmic Microtubule Organization	9.636
37	Response To Starvation	9.417
38	Negative Regulation Of Protein Complex Assembly	9.256
39	Histone H3-K9 Demethylation	9.256
40	Axon Extension	8.985
41	Establishment Of Endothelial Barrier	8.678
42	Negative Regulation Of Collateral Sprouting	8.343
43	Protein K63-Linked Deubiquitination	8.343
44	Microtubule Severing	8.343
45	Plus-End-Directed Vesicle Transport Along Microtubule	8.343
46	Insulin Secretion Involved In Cellular Response To Glucose Stimulus	8.343
47	Positive Regulation Of Receptor Biosynthetic Process	8.343
48	Negative Regulation Of Interferon-Gamma-Mediated Signaling Pathway	8.343
49	Regulation Of Protein Export From Nucleus	8.343
50	Regulation Of Circadian Sleep/Wake Cycle, Sleep	8.343
51	Regulation Of Cellular Amino Acid Metabolic Process	8.343

52	Autophagosome Assembly	8.234
53	Autophagy	8.22
54	Microtubule Nucleation	7.308
55	Platelet Formation	7.308
56	Neuron Projection Regeneration	7.3
57	Negative Regulation Of B Cell Apoptotic Process	7.3
58	Regulation Of Dendritic Spine Development	7.3
59	Cellular Response To Testosterone Stimulus	7.3
60	Negative Regulation Of Type I Interferon-Mediated Signaling Pathway	7.3
61	Entry Of Bacterium Into Host Cell	7.3
62	Death-Inducing Signaling Complex Assembly	7.3
63	Protein Targeting To Mitochondrion	6.871
64	Establishment Of Protein Localization To Membrane	6.489
65	Histone H2B Ubiquitination	6.489
66	Regulation Of Glucose Import	6.489
67	Dephosphorylation	6.376
68	Phosphatidylinositol Dephosphorylation	6.311
69	Entrainment Of Circadian Clock By Photoperiod	6.311
70	Circadian Regulation Of Gene Expression	6.164
71	Endosome Organization	6.147
72	Establishment Of Mitotic Spindle Orientation	6.037
73	Deadenylation-Dependent Decapping Of Nuclear-Transcribed Mrna	5.84
74	Intraciliary Transport Involved In Cilium Assembly	5.84
75	Response To Uv-C	5.84
76	Histone H3-K36 Demethylation	5.84
77	Positive Regulation Of Transcription, Dna-Templated	5.8
78	Negative Regulation Of Microtubule Depolymerization	5.785
79	Positive Regulation Of Extrinsic Apoptotic Signaling Pathway	5.785
80	Protein Autophosphorylation	5.774
81	Negative Regulation Of Neuron Differentiation	5.65
82	Regulation Of Megakaryocyte Differentiation	5.562
83	Fatty Acid Metabolic Process	5.557
84	Protein Phosphorylation	5.359
85	Steroid Biosynthetic Process	5.34
86	Microtubule Bundle Formation	5.34
87	Cellular Response To Insulin Stimulus	5.328
88	Cellular Response To Nutrient Levels	5.309
89	Positive Regulation Of Autophagosome Assembly	5.309
90	Rap Protein Signal Transduction	5.309
91	Positive Regulation Of Nuclear-Transcribed Mrna Poly(A) Tail Shortening	5.309
92	Cell-Cell Junction Assembly	5.309
93	Cyclooxygenase Pathway	5.309
94	Gene Silencing By Mirna	5.309
95	Regulation Of Stem Cell Population Maintenance	5.309
96	Mitochondrial Transcription	5.309
97	Transcription By Rna Polymerase Ii	5.302
98	Cholesterol Metabolic Process	5.215
99	Macroautophagy	5.206
100	Activation Of Protein Kinase Activity	4.977
101	Protein Deubiquitination	4.965
102	Regulation Of Tumor Necrosis Factor-Mediated Signaling Pathway	4.959
103	Regulation Of Circadian Rhythm	4.867
104	Histone Monoubiquitination	4.867
105	White Fat Cell Differentiation	4.867
106	Mrna Cleavage	4.867

107	Regulation Of Histone Methylation	4.867
108	Negative Regulation By Host Of Viral Transcription	4.867
109	Regulation Of Protein Stability	4.843
110	Neuron Projection Development	4.782
111	Protein K48-Linked Deubiquitination	4.628
112	Regulation Of Gene Expression	4.591
113	Cellular Response To Hormone Stimulus	4.58
114	Cellular Response To Cholesterol	4.492
115	Peptidyl-Serine Phosphorylation	4.492
116	Protein Neddylation	4.492
117	Protein Localization To Phagophore Assembly Site	4.492
118	Covalent Chromatin Modification	4.492
119	Wnt Signaling Pathway	4.479
120	Cellular Response To Dexamethasone Stimulus	4.479
121	Positive Regulation Of Transcription By Rna Polymerase Ii	4.447
122	Cellular Response To Camp	4.408
123	Positive Regulation Of Protein Kinase Activity	4.408
124	Positive Regulation Of Neuron Differentiation	4.346
125	Anterograde Axonal Transport	4.339
126	Positive Regulation Of Gene Expression, Epigenetic	4.339
127	Negative Regulation Of Insulin Receptor Signaling Pathway	4.339
128	Glucose Homeostasis	4.313
129	Cell Proliferation	4.241
130	Nucleobase-Containing Compound Metabolic Process	4.207
131	3'-Utr-Mediated Mrna Destabilization	4.171
132	Positive Regulation Of Nuclear-Transcribed Mrna Catabolic Process, Deadenylation-Dependent Decay	4.171
133	Prostaglandin Biosynthetic Process	4.171
134	Protein Polyubiquitination	4.145
135	Spermatid Development	4.134
136	Locomotory Behavior	4.134
137	Cell Death	4.084
138	Response To Nutrient Levels	4.084
139	Positive Regulation Of Apoptotic Process	4.079
140	In Utero Embryonic Development	4.074
141	Oxidation-Reduction Process	3.985
142	Brain Development	3.967
143	Positive Regulation Of Epithelial Cell Migration	3.967
144	Negative Regulation Of Extrinsic Apoptotic Signaling Pathway Via Death Domain Receptors	3.967
145	Cellular Response To Glucose Stimulus	3.959
146	Protein Dephosphorylation	3.93
147	Neuromuscular Process	3.893
148	Positive Regulation Of Keratinocyte Differentiation	3.893
149	Negative Regulation Of Neurogenesis	3.893
150	Megakaryocyte Development	3.893
151	Chromosome Organization	3.893
152	Histone Mrna Catabolic Process	3.893
153	Endocytosis	3.877
154	Trna Aminoacylation For Protein Translation	3.857
155	Positive Regulation Of Gtpase Activity	3.772
156	Negative Regulation Of Transcription By Rna Polymerase Ii	3.769
157	G2/M Transition Of Mitotic Cell Cycle	3.743
158	Protein Ubiquitination	3.72
159	Response To Estrogen	3.708
160	Regulation Of Mitotic Cell Cycle	3.685
161	Phosphatidic Acid Biosynthetic Process	3.654

162	Regulation Of Macroautophagy	3.65
163	Vascular Endothelial Growth Factor Receptor Signaling Pathway	3.65
164	Stimulatory C-Type Lectin Receptor Signaling Pathway	3.65
165	Alternative Mrna Splicing, Via Spliceosome	3.65
166	Signal Transduction	3.646
167	Smoothened Signaling Pathway	3.594
168	Protein Destabilization	3.471
169	Intracellular Receptor Signaling Pathway	3.471
170	Cellular Response To Epidermal Growth Factor Stimulus	3.471
171	Protein Localization To Plasma Membrane	3.457
172	Positive Regulation Of Histone Acetylation	3.435
173	Ras Protein Signal Transduction	3.435
174	Camera-Type Eye Morphogenesis	3.435
175	Negative Regulation Of Transcription, Dna-Templated	3.429
176	Cellular Response To Glucose Starvation	3.386
177	Transcription Initiation From Rna Polymerase Ii Promoter	3.336
178	Phosphorylation	3.324
179	Chromatin Remodeling	3.324
180	Regulation Of Cilium Assembly	3.306
181	Non-Motile Cilium Assembly	3.306
182	Regulation Of Actin Cytoskeleton Reorganization	3.244
183	Chromosome Segregation	3.244
184	Apoptotic Cell Clearance	3.244
185	Positive Regulation Of Transcription Of Notch Receptor Target	3.244
186	Regulation Of Neuron Apoptotic Process	3.244
187	3'-Utr-Mediated Mrna Stabilization	3.244
188	Regulation Of Cellular Response To Heat	3.229
189	Regulation Of Synaptic Plasticity	3.156
190	Transcription, Dna-Templated	3.132
191	Response To Toxic Substance	3.115
192	Actin Cytoskeleton Organization	3.102
193	Regulation Of Catalytic Activity	3.085
194	Endoplasmic Reticulum Unfolded Protein Response	3.085
195	Mrna Stabilization	3.074
196	Histone H3 Deacetylation	3.074
197	Negative Regulation Of Rho Protein Signal Transduction	3.074
198	Triglyceride Biosynthetic Process	3.074
199	Protein Localization To Golgi Apparatus	3.074
200	Multicellular Organism Development	3.056
201	Mapk Cascade	3.047
202	Ubiquitin-Dependent Protein Catabolic Process	3.021
203	Rna Secondary Structure Unwinding	3.018
204	Androgen Receptor Signaling Pathway	3.018
205	Cellular Response To Cytokine Stimulus	3.018
206	Heart Development	2.958
207	Positive Regulation Of Stress Fiber Assembly	2.954
208	Cell Cycle	2.954
209	Dna Duplex Unwinding	2.954
210	Skeletal Muscle Tissue Development	2.954
211	Negative Regulation Of Inflammatory Response	2.92
212	Positive Regulation Of Protein Export From Nucleus	2.92
213	Circadian Rhythm	2.92
214	Negative Regulation Of Stress Fiber Assembly	2.92
215	Protein Import Into Mitochondrial Matrix	2.92
216	Neurotrophin Trk Receptor Signaling Pathway	2.92

217	Nervous System Development	2.904
218	Double-Strand Break Repair Via Homologous Recombination	2.884
219	Insulin Receptor Signaling Pathway	2.849
220	Regulation Of G2/M Transition Of Mitotic Cell Cycle	2.849
221	Protein Stabilization	2.813
222	Regulation Of Mitotic Spindle Assembly	2.781
223	Rac Protein Signal Transduction	2.781
224	B Cell Homeostasis	2.781
225	Negative Regulation Of Protein Serine/Threonine Kinase Activity	2.781
226	Response To Light Stimulus	2.781
227	Positive Regulation Of Intracellular Protein Transport	2.781
228	Negative Regulation Of Gene Expression	2.778
229	Phosphatidylinositol-3-Phosphate Biosynthetic Process	2.777
230	Cell Division	2.74
231	Negative Regulation Of Canonical Wnt Signaling Pathway	2.734
232	Regulation Of Vesicle Fusion	2.722
233	Somatic Stem Cell Population Maintenance	2.722
234	Regulation Of Transcription, Dna-Templated	2.679
235	Nucleotide-Binding Oligomerization Domain Containing Signaling Pathway	2.655
236	Telomere Maintenance Via Telomerase	2.655
237	Melanosome Organization	2.655
238	Mrna Processing	2.633
239	Regulation Of Lipid Metabolic Process	2.625
240	Positive Regulation Of Smooth Muscle Cell Proliferation	2.62
241	Hemopoiesis	2.571
242	Negative Regulation Of Catalytic Activity	2.571
243	Cell Morphogenesis	2.567
244	Activation Of Gtpase Activity	2.539
245	Cellular Response To Glucocorticoid Stimulus	2.539
246	Dna Methylation	2.539
247	Lipid Metabolic Process	2.53
248	Protein Autoubiquitination	2.524
249	Small Gtpase Mediated Signal Transduction	2.512
250	Cellular Response To Drug	2.479
251	Defense Response To Virus	2.475
252	Ciliary Basal Body-Plasma Membrane Docking	2.459
253	Signal Transduction By Protein Phosphorylation	2.436
254	Positive Regulation Of Defense Response To Virus By Host	2.433
255	Mrna Export From Nucleus	2.408
256	Regulation Of Small Gtpase Mediated Signal Transduction	2.404
257	Heart Looping	2.394
258	Intracellular Signal Transduction	2.36
259	Regulation Of Transcription By Rna Polymerase Ii	2.329
260	Apoptotic Process	2.297
261	Double-Strand Break Repair	2.276
262	Neuron Migration	2.268
263	Negative Regulation Of Protein Kinase Activity	2.246
264	Neuron Projection Morphogenesis	2.239
265	Mitotic Cytokinesis	2.239
266	Regulation Of Translation	2.239
267	Regulation Of Alternative Mrna Splicing, Via Spliceosome	2.239
268	Cilium Assembly	2.22
269	Intracellular Protein Transport	2.204
270	Regulation Of Cell Cycle	2.204
271	Protein Homooligomerization	2.199

272	Regulation Of Insulin Secretion	2.169
273	Positive Regulation Of Protein Binding	2.169
274	Phospholipid Biosynthetic Process	2.163
275	Regulation Of Transcription From Rna Polymerase Ii Promoter In Response To Hypoxia	2.163
276	Pituitary Gland Development	2.163
277	I-Kappab Kinase/Nf-Kappab Signaling	2.136
278	Peptidyl-Tyrosine Phosphorylation	2.132
279	Dna Repair	2.106
280	Adipose Tissue Development	2.086
281	Synaptic Transmission, Glutamatergic	2.086
282	Bile Acid And Bile Salt Transport	2.086
283	Limb Development	2.086
284	Endocytic Recycling	2.086
285	Protein Homotrimerization	2.086
286	Negative Regulation Of Insulin Secretion	2.086
287	Golgi To Plasma Membrane Protein Transport	2.086
288	Regulation Of Cell Shape	2.08
289	Phosphatidylinositol Phosphorylation	2.067
290	Protein-Containing Complex Assembly	2.031
291	Active Evasion Of Host Immune Response Via Regulation Of Host Antigen Processing And Presentation	2.014
292	Beta-Catenin-Tcf Complex Assembly	2.014
293	Negative Regulation Of Type I Interferon Production	2.014
294	Endothelial Cell Migration	2.014
295	Positive Regulation Of Protein Import Into Nucleus	2.014
296	Neuromuscular Junction Development	2.014
297	Glycogen Metabolic Process	2.014
298	Cell Differentiation	1.997
299	Notch Signaling Pathway	1.997
300	Activation Of Mapk Activity	1.997
301	Cellular Response To Dna Damage Stimulus	1.986
302	Regulation Of Gtpase Activity	1.983
303	Regulation Of Mitochondrial Membrane Potential	1.947
304	Positive Regulation Of Tor Signaling	1.947
305	Positive Regulation Of Smoothened Signaling Pathway	1.947
306	Hippo Signaling	1.947
307	Extrinsic Apoptotic Signaling Pathway In Absence Of Ligand	1.947
308	Rab Protein Signal Transduction	1.928
309	Positive Regulation Of Gene Expression	1.919
310	Positive Regulation Of Glucose Import	1.884
311	Rna Phosphodiester Bond Hydrolysis, Exonucleolytic	1.884
312	Dna Damage Checkpoint	1.884
313	Behavioral Fear Response	1.884
314	Dorsal/Ventral Pattern Formation	1.884
315	Positive Regulation Of Mitotic Cell Cycle	1.884
316	Peptidyl-Threonine Phosphorylation	1.876
317	Response To Endoplasmic Reticulum Stress	1.876
318	Dna Recombination	1.876
319	Protein Transport	1.844
320	Positive Regulation Of Proteasomal Ubiquitin-Dependent Protein Catabolic Process	1.827
321	Lipid Homeostasis	1.825
322	Regulation Of Rna Splicing	1.825
323	Mrna Splicing, Via Spliceosome	1.819
324	Cellular Response To Calcium Ion	1.803
325	Negative Regulation Of Cell Proliferation	1.783
326	Endosomal Transport	1.78

327	Fibroblast Growth Factor Receptor Signaling Pathway	1.78
328	Negative Regulation Of Tor Signaling	1.77
329	Histone H4 Acetylation	1.77
330	Exonucleolytic Nuclear-Transcribed Mrna Catabolic Process Involved In Deadenylation-Dependent Decay	1.77
331	Positive Regulation Of Telomerase Activity	1.718
332	Stress-Activated Protein Kinase Signaling Cascade	1.718
333	Liver Regeneration	1.718
334	Multicellular Organism Growth	1.714
335	Regulation Of Signal Transduction By P53 Class Mediator	1.714
336	Sensory Perception Of Sound	1.681
337	Positive Regulation Of Peptidyl-Serine Phosphorylation	1.673
338	Rho Protein Signal Transduction	1.673
339	Long-Term Memory	1.669
340	Cellular Response To Estradiol Stimulus	1.669
341	Post-Translational Protein Modification	1.668
342	Positive Regulation Of Nf-Kappab Transcription Factor Activity	1.645
343	Sodium Ion Transport	1.633
344	Response To Organic Substance	1.622
345	Semaphorin-Plexin Signaling Pathway	1.622
346	Histone Acetylation	1.622
347	Dendrite Morphogenesis	1.622
348	Regulation Of Apoptotic Process	1.584
349	Positive Regulation Of Jun Kinase Activity	1.578
350	Hair Follicle Development	1.578
351	Cell Migration	1.555
352	Positive Regulation Of Protein Phosphorylation	1.547
353	Glycosaminoglycan Biosynthetic Process	1.537
354	Negative Regulation Of G0 To G1 Transition	1.537
355	Cellular Response To Leukemia Inhibitory Factor	1.526
356	Male Gonad Development	1.509
357	Fertilization	1.497
358	Chondrocyte Differentiation	1.497
359	Sodium Ion Transmembrane Transport	1.493
360	Negative Regulation Of Signal Transduction	1.477
361	Angiogenesis	1.474
362	Positive Regulation Of Erk1 And Erk2 Cascade	1.474
363	Regulation Of Endocytosis	1.46
364	Regulation Of Cytokinesis	1.46
365	Scf-Dependent Proteasomal Ubiquitin-Dependent Protein Catabolic Process	1.46
366	Spermatogenesis	1.453
367	Transmembrane Transport	1.436
368	Cytoskeleton Organization	1.433
369	Positive Regulation Of B Cell Proliferation	1.424
370	Steroid Metabolic Process	1.424
371	Negative Regulation Of Autophagy	1.424
372	Positive Regulation Of Autophagy	1.424
373	Negative Regulation Of Protein Kinase B Signaling	1.424
374	Autophagy Of Mitochondrion	1.424
375	Bone Development	1.39
376	Erythrocyte Differentiation	1.39
377	Actin Filament Bundle Assembly	1.39
378	Rna Splicing	1.374
379	Positive Regulation Of Insulin Secretion	1.358
380	Phosphatidylinositol-Mediated Signaling	1.358
381	Viral Process	1.348

382	Platelet Activation	1.335
383	Histone H3 Acetylation	1.327
384	Activation Of Mapkk Activity	1.327
385	Rna Metabolic Process	1.327
386	Anatomical Structure Development	1.298
387	Endosome To Lysosome Transport	1.298
388	Inner Ear Development	1.298
389	Negative Regulation Of Neuron Death	1.298
390	Cell-Cell Adhesion	1.298
391	Positive Regulation Of Protein Serine/Threonine Kinase Activity	1.27
392	Calcium-Dependent Cell-Cell Adhesion Via Plasma Membrane Cell Adhesion Molecules	1.27
393	Response To Organic Cyclic Compound	1.27
394	Positive Regulation Of Dna Binding Transcription Factor Activity	1.262
395	Positive Regulation Of Cysteine-Type Endopeptidase Activity Involved In Apoptotic Process	1.243
396	Inositol Phosphate Metabolic Process	1.243
397	Gluconeogenesis	1.243
398	Negative Regulation Of Protein Ubiquitination	1.243
399	Regulation Of Signal Transduction	1.243
400	Histone Deacetylation	1.217
401	Microtubule-Based Movement	1.197
402	Neutrophil Degranulation	1.193
403	Positive Regulation Of Fat Cell Differentiation	1.192
404	Response To Cytokine	1.192
405	Receptor-Mediated Endocytosis	1.18
406	Calcium Ion Transmembrane Transport	1.177
407	Positive Regulation Of Nik/Nf-Kappab Signaling	1.168
408	Learning Or Memory	1.168
409	Phagocytosis	1.168
410	Positive Regulation Of Mapk Cascade	1.147
411	Interleukin-1-Mediated Signaling Pathway	1.145
412	Somitogenesis	1.145
413	Cellular Response To Uv	1.145
414	T Cell Costimulation	1.145
415	Spindle Assembly	1.145
416	Cellular Response To Tumor Necrosis Factor	1.138
417	Steroid Hormone Mediated Signaling Pathway	1.102
418	Dna Replication	1.093
419	Proteolysis	1.093
420	Cellular Response To Transforming Growth Factor Beta Stimulus	1.081
421	Regulation Of Mapk Cascade	1.081
422	Chromatin Organization	1.076
423	Negative Regulation Of Phosphatase Activity	1.062
424	Mrna 3'-End Processing	1.062
425	Cell-Cell Signaling	1.046
426	Glucose Metabolic Process	1.043
427	Dna Damage Response, Signal Transduction By P53 Class Mediator Resulting In Cell Cycle Arrest	1.043
428	Retinoid Metabolic Process	1.025
429	Negative Regulation Of Epithelial Cell Proliferation	1.025
430	Embryonic Digit Morphogenesis	1.007
431	Cartilage Development	1.007

Table S4. KEGG enrichment from RNA-seq DEGs

	KEGG pathway	Enrichment score
1	Steroid Biosynthesis	42.482
2	Terpenoid Backbone Biosynthesis	17.748
3	Longevity Regulating Pathway	12.553
4	Renal Cell Carcinoma	9.578
5	Ampk Signaling Pathway	9.31
6	Inositol Phosphate Metabolism	8.931
7	Circadian Rhythm	8.679
8	Parathyroid Hormone Synthesis, Secretion And Action	7.615
9	Phosphatidylinositol Signaling System	6.676
10	Autophagy - Animal	6.306
11	Mitophagy - Animal	6.007
12	Hedgehog Signaling Pathway	5.725
13	Thyroid Hormone Signaling Pathway	5.698
14	Adipocytokine Signaling Pathway	5.659
15	Notch Signaling Pathway	5.605
16	Neurotrophin Signaling Pathway	5.554
17	Adherens Junction	5.423
18	Bacterial Invasion Of Epithelial Cells	5.276
19	Metabolic Pathways	5.187
20	Glucagon Signaling Pathway	5.064
21	Insulin Resistance	4.875
22	Viral Carcinogenesis	4.774
23	Vegf Signaling Pathway	4.56
24	Herpes Simplex Infection	4.363
25	Central Carbon Metabolism In Cancer	4.139
26	Focal Adhesion	4.056
27	Long-Term Potentiation	4.016
28	Foxo Signaling Pathway	3.952
29	Tight Junction	3.888
30	Estrogen Signaling Pathway	3.807
31	Mapk Signaling Pathway	3.787
32	Vasopressin-Regulated Water Reabsorption	3.634
33	Leukocyte Transendothelial Migration	3.486
34	Mtor Signaling Pathway	3.454
35	Oxytocin Signaling Pathway	3.432
36	Pathways In Cancer	3.408
37	Egfr Tyrosine Kinase Inhibitor Resistance	3.406
38	Camp Signaling Pathway	3.338
39	Sphingolipid Signaling Pathway	3.309
40	Proteoglycans In Cancer	3.288
41	Vascular Smooth Muscle Contraction	3.227
42	Rap1 Signaling Pathway	3.208
43	Vibrio Cholerae Infection	3.198
44	Human Cytomegalovirus Infection	2.937
45	Pathogenic Escherichia Coli Infection	2.908
46	Apoptosis	2.871
47	Insulin Signaling Pathway	2.85
48	Apelin Signaling Pathway	2.85
49	Ras Signaling Pathway	2.849
50	Human Papillomavirus Infection	2.83
51	Fluid Shear Stress And Atherosclerosis	2.809
52	Transcriptional Misregulation In Cancer	2.804

53	Prostate Cancer	2.774
54	Glycerophospholipid Metabolism	2.774
55	Endometrial Cancer	2.757
56	Endocrine Resistance	2.745
57	Phospholipase D Signaling Pathway	2.674
58	Melanogenesis	2.664
59	Breast Cancer	2.656
60	C-Type Lectin Receptor Signaling Pathway	2.587
61	Longevity Regulating Pathway - Multiple Species	2.579
62	Regulation Of Actin Cytoskeleton	2.449
63	Tnf Signaling Pathway	2.446
64	Acute Myeloid Leukemia	2.423
65	Butanoate Metabolism	2.402
66	Cgmp-Pkg Signaling Pathway	2.395
67	Epithelial Cell Signaling In Helicobacter Pylori Infection	2.352
68	Prolactin Signaling Pathway	2.284
69	Thermogenesis	2.278
70	Axon Guidance	2.231
71	Melanoma	2.221
72	Cell Cycle	2.17
73	Alcoholism	2.169
74	Endocytosis	2.138
75	Chronic Myeloid Leukemia	2.104
76	Relaxin Signaling Pathway	2.07
77	Chemokine Signaling Pathway	2.066
78	Dopaminergic Synapse	2.054
79	Spliceosome	2.008
80	Epstein-Barr Virus Infection	1.943
81	Peroxisome	1.927
82	Erbb Signaling Pathway	1.881
83	Adrenergic Signaling In Cardiomyocytes	1.868
84	Hepatitis B	1.868
85	Human Immunodeficiency Virus 1 Infection	1.842
86	Gap Junction	1.817
87	Non-Alcoholic Fatty Liver Disease (Nafld)	1.806
88	Gastric Cancer	1.806
89	Mrna Surveillance Pathway	1.757
90	Cushing Syndrome	1.747
91	Hippo Signaling Pathway	1.747
92	Micrornas In Cancer	1.745
93	Gnrh Signaling Pathway	1.72
94	Il-17 Signaling Pathway	1.72
95	Glycine, Serine And Threonine Metabolism	1.682
96	Jak-Stat Signaling Pathway	1.661
97	Bladder Cancer	1.641
98	Nod-Like Receptor Signaling Pathway	1.602
99	Hepatocellular Carcinoma	1.602
100	T Cell Receptor Signaling Pathway	1.583
101	Influenza A	1.573
102	Abc Transporters	1.529
103	Basal Transcription Factors	1.495
104	Pi3K-Akt Signaling Pathway	1.473
105	Kaposi Sarcoma-Associated Herpesvirus Infection	1.447
106	Valine, Leucine And Isoleucine Degradation	1.401
107	Amino Sugar And Nucleotide Sugar Metabolism	1.401

108	Huntington Disease	1.394
109	Cocaine Addiction	1.373
110	Mineral Absorption	1.319
111	Amyotrophic Lateral Sclerosis (Als)	1.319
112	Platelet Activation	1.3
113	Osteoclast Differentiation	1.249
114	Hepatitis C	1.221
115	Natural Killer Cell Mediated Cytotoxicity	1.221
116	Steroid Hormone Biosynthesis	1.14
117	Lysine Degradation	1.14
118	Wnt Signaling Pathway	1.095
119	Arachidonic Acid Metabolism	1.068
120	Cortisol Synthesis And Secretion	1.051
121	Non-Small Cell Lung Cancer	1.019
122	Aminoacyl-Trna Biosynthesis	1.019

Table S5. GO enrichment from LC-MS/MS DEPs

	Gene Ontology Term (GO term)	Enrichment score
1	Retrograde Protein Transport, Er To Cytosol	92.73
2	Positive Regulation Of Glycoprotein Metabolic Process	92.73
3	Vesicle Transport Along Microtubule	49.92
4	Triglyceride Mobilization	46.36
5	Interleukin-8 Secretion	46.36
6	Regulation Of Protein Metabolic Process	30.91
7	Protein C-Linked Glycosylation Via 2'-Alpha-Mannosyl-L-Tryptophan	26.49
8	Growth Hormone Secretion	26.49
9	Leukotriene Biosynthetic Process	18.55
10	Cellular Response To Insulin Stimulus	17.45
11	Virion Assembly	16.86
12	Cargo Loading Into Copii-Coated Vesicle	14.27
13	Positive Regulation Of Ubiquitin Protein Ligase Activity	14.27
14	Establishment Of Endothelial Intestinal Barrier	14.27
15	Er-Associated Misfolded Protein Catabolic Process	14.27
16	Phagocytosis	13.98
17	Lipoprotein Transport	13.25
18	Erad Pathway	13.25
19	Ubiquitin-Dependent Erad Pathway	10.43
20	Regulation Of Actin Cytoskeleton Organization	10.28
21	Rab Protein Signal Transduction	9.71
22	Error-Free Translesion Synthesis	9.27
23	Protein Folding	9.05
24	Melanosome Transport	8.43
25	Proteasome-Mediated Ubiquitin-Dependent Protein Catabolic Process	8.15
26	Intracellular Protein Transport	7.86
27	Neutrophil Degranulation	7.73
28	Receptor-Mediated Endocytosis	7.49
29	Er To Golgi Vesicle-Mediated Transport	7.31
30	Post-Translational Protein Modification	7.27
31	Regulation Of Cell Morphogenesis	7.13
32	Osteoblast Differentiation	6.92
33	Regulation Of Cell Motility	6.62
34	Response To Virus	6.59
35	Protein Methylation	6.18
36	Autophagy	5.92
37	Golgi Organization	5.87
38	Protein Deubiquitination	5.86
39	Atp Metabolic Process	5.8
40	Proteasomal Protein Catabolic Process	5.45
41	Actin Cytoskeleton Organization	4.79
42	Substrate Adhesion-Dependent Cell Spreading	4.52
43	Response To Lipopolysaccharide	4.29
44	Biological_Process	4.01
45	Translation	3.7
46	Autophagosome Assembly	3.37
47	Retinoid Metabolic Process	3.25
48	Negative Regulation Of Neuron Projection Development	3.25
49	Cerebral Cortex Development	3.14
50	Antimicrobial Humoral Immune Response Mediated By Antimicrobial Peptide	3.09
51	Copii Vesicle Coating	2.94

52	Positive Regulation Of Protein Catabolic Process	2.51
53	Response To Endoplasmic Reticulum Stress	2.51
54	Lipid Catabolic Process	2.23
55	Rho Protein Signal Transduction	2.23
56	Retrograde Vesicle-Mediated Transport, Golgi To Er	2.21
57	Actin Filament Organization	1.77
58	Vesicle-Mediated Transport	1.57
59	Cellular Response To Tumor Necrosis Factor	1.52
60	Platelet Degranulation	1.5
61	Protein Phosphorylation	1.48
62	Negative Regulation Of Neuron Apoptotic Process	1.43
63	Positive Regulation Of Cell Proliferation	1.4
64	Lipid Metabolic Process	1.38
65	Oxidation-Reduction Process	1.27
66	Endocytosis	1.25
67	Aging	1.17
68	Cytoskeleton Organization	1.14

Table S6. KEGG enrichment from LC-MS/MS DEPs

	**KEGG pathway**	**Enrichment score**
1	Protein Processing In Endoplasmic Reticulum	7.805
2	Legionellosis	7.178
3	Vitamin Digestion And Absorption	6.919
4	Fat Digestion And Absorption	4.05
5	Proteasome	3.69
6	Ether Lipid Metabolism	3.533
7	Amino Sugar And Nucleotide Sugar Metabolism	3.46
8	Cholesterol Metabolism	3.321
9	Phagosome	2.597
10	Aminoacyl-Trna Biosynthesis	2.516
11	Metabolic Pathways	2.444
12	Ppar Signaling Pathway	2.244
13	Amoebiasis	1.73
14	Glycerophospholipid Metabolism	1.712
15	Insulin Resistance	1.552
16	Vascular Smooth Muscle Contraction	1.372
17	Systemic Lupus Erythematosus	1.249
18	Spliceosome	1.239

Table S7. List of qPCR primers

Species	Gene	Forward primer (5'-3')	Reverse primer (5'-3')
D. rerio	*atg101*	TCACTTTCGTGCGAGTGTCT	CACTCGTCTGAAAAGGGCCA
	bnip4	CAGTCCCAGACGTGTCCAGA	GATCCTTGCAACAGCTTCGG
	fabp10a	AACTCCTTCACCATCGGCAA	ATCATGGTGGTTCCTCCGAC
	fdps	TGAGCCAGGACGAGTCCATA	CCTCCAGGGGCATTGTACTG
	lsm12b	CGTCGTAATCTCACCACCGT	TCCTTCTGTGTTTGCTGTGC
	lss	GGCATTTGCGTGTATGGGTC	ATCAATCCCAGCAAAGCCCA
	map1lc3a	ACCCTTCAAACAACGACGGA	TGGAAGTTGCTTTTCCCCCT
	mvk	GAGCATTCTGTCGTGCATGG	AAGTTCCTTGGGGTCCCTCT
	rpl13a	ATCATGGCGGACCGATTCAA	TCTTGCGGAGGAAAGCCAAA
H. sapiens	*ACTB*	ACCATGGATGATGATATCGC	CATAGGAATCCTTCTGACCCA
	FDPS	GGTAGTAGCATTCCGGGAG	ATTGATGGCATCCAAACCCA
	HMGCR	TAGCTGGACGCAACCTTTAT	TAGCAGCAGGTTTCTTGTCA
	HMGCS	ACCGCTGCTATTCTGTCTAC	GTCATTCAGCAACATCCGAG
	LSS	GAGCGGCGTTATTTGCAGAG	AGACACCGGACTCCTCTCTC
	MVK	TTCCCAGGAGCCATGTTGTC	TACAGCCAGTGCTACCTTGC
	TBP	GTTCAGCAGTCAACGTCCCA	TCATGGGGGAGGGATACAGT

Adjusted weighted average of gene expression within KEGG pathway nodes

The weighted single gene is calculated taking the relative expression level of a gene (Z:A, $\log_2$) and multiplying it by the complement of the statistical significance of the relative expression (1 - p-value). Using this method, gene with a less statistically significant difference has a higher p-value which when complemented will be closer to zero and when multiplied by the gene expression level will result in a lower magnitude weighted gene expression. A gene with more statistically significant difference has a lower p-value which when complemented will be closer to one, which will result in less changed magnitude weighted gene expression. Overall, this will decrease the perceived influence of less significant genes whereas more significant genes remain more unchanged. A weighting power is applied to increase the weighting given to the statistical significance.

$$Weighted\ single\ gene\ =\ gene\ expression \times (1 - significance)^{weight}$$

Example (weight of 5):

Gene	Expression (Z:A, Log2)	Significance (Z:A, p-value)	Weighted single gene (Z:A)
lmnb1	-0.60	0.27	-0.12
lmna	-0.90	0.00	-0.89
lmnb2	0.20	0.87	0.00

In signalling pathway networks, individual nodes often contain multiple genes that contribute to the node as each gene performs the function of the node. The one-to-many relationship of human to zebrafish orthologs exacerbates the number of genes in nodes. To apply the weighting methodology to multiple genes within a node, a weighted averaged is calculated. The weighted single gene expressions are summed and divided by the sum of the significance to calculate the weighted multiple gene.

$$Weighted\ multiple\ gene = \frac{\sum_{node\ genes}(gene\ expression \times (1 - significance)^{weight})}{\sum_{node\ genes}((1 - significance)^{weight})}$$

One limitation of a weighted average is nodes where all genes have little significance can be misleading as the denominator remains small, so the weighting on significance is lost. This is adjusted for my multiplying the weighted multiple gene by the complement of the product of all the genes' significance - that is the overall significance of the node. Again, this significance is given a weighting power.

$$Adjusted\ weighted\ multiple\ gene = \frac{\sum_{node\ genes}(gene\ expression \times (1 - significance)^{weight})}{\sum_{node\ genes}((1 - significance)^{weight})} \times (1 - \prod_{node\ genes} significance)^{weight}$$
